# Rheological properties of transglutaminase-treated concentrated pea protein under conditions relevant to high-moisture extrusion processing

**DOI:** 10.3389/fnut.2022.970010

**Published:** 2022-08-12

**Authors:** Jianxin Qin, Yinghan Zhao, Jingwen Zhou, Guoqiang Zhang, Jianghua Li, Xiao Liu

**Affiliations:** ^1^Science Center for Future Foods, Jiangnan University, Wuxi, China; ^2^National Engineering Laboratory for Cereal Fermentation Technology, Jiangnan University, Wuxi, China

**Keywords:** transglutaminase, pea protein, rheological properties, closed cavity rheometer, high-moisture extrusion

## Abstract

At present, the structural changes of extruded materials under thermal-mechanical stress during high-moisture extrusion are still unclear. In this study, the transglutaminase (TG) treatments on the structure of pea protein isolate (PPI) under conditions relevant to high-moisture extrusion processing (50 wt% PPI at 30°C, 120°C and heated to 120°C and subsequently cooled to 30°C) was studied by using a closed cavity rheometer. Strain and frequency sweeping were carried out under various temperature conditions, and the information obtained was drawn into a texture map. Lissajous curves combined with energy dissipation ratio were introduced to characterize the nonlinear response of the samples. The results showed that the storage modulus of PPI increased with the increase of TG concentration during heat treatment. After cooling to 30°C, PPI with 0.25–1%TG could enhance the elasticity, but treating by 2% TG could inhibit the formation of disulfide bonds, the uniform development of the protein network, thus showing the “tough” character. These findings can help to better understand the relationships of material-structure during the extrusion process, and also provide help for further optimization of the quality of meat substitutes.

## Introduction

The rapid growth of the world’s population has led to a shortage of meat, and plant-based meat has gradually begun to be commercially produced because it is more efficient and environmentally sustainable (1–3). Texture is an important index to evaluate the quality of plant-based meat, which helps to improve the acceptance of consumers (4). The formation of texture during food processing can be partially improved by adding different components, such as proteins, polysaccharides and crosslinking agents (5). As a crosslinking enzyme, Transglutaminase (TG) can cross-link glutamine residues with lysine to form a ε-(γ-glutamyl) lysine bonds to change the functional properties of proteins, thus contributing to the quality of gel structure of foods (6, 7).

High-moisture extrusion is a promising process to create the fiber structure of plant-based meat. Previous studies have reported that TG modification can improve the texture of high-moisture extrudates (8, 9). However, the extrusion process is regarded as a “black box” process, and the setting of conditions and the production of extrudates depend on operating experience (10, 11). The sudden stop sampling method is a common method to study the state of the sample during extrusion (12–14). Unfortunately, this method cannot systematically study the structural changes of extrudates under thermal-mechanical stress during high-moisture extrusion.

Recently, a closed cavity rheometer has been used to investigate the rheological properties of extrudates during extrusion (15–17). The device combines the advantages of the traditional rheometer and shear cell, which can not only carry out thermo-mechanical treatment of samples under high pressure and sealed conditions, but also measure the rheological properties of samples online (18). Additionally, large amplitude oscillatory shear (LAOS) of protein samples combined with Lissajous curves can provide additional information (19, 20), which is helpful to simulate the structural changes and rheological behavior of protein materials during extrusion, leading to solving the problem of “black box” process to a great extent.

Pea protein is a high-quality protein resource with a relatively balanced amino acid content. Consumers are more interested in plant-based foods made from pea protein (21, 22). More importantly, pea protein is more acceptable because it has no allergenic effect. Therefore, in this study, pea protein isolate (PPI) was invoked as a model, and the structural changes of PPI treated with TG during high-moisture extrusion were investigated by a closed cavity rheometer. Strain and frequency sweep were carried out under various temperature conditions, and Lissajous curve was introduced to characterize the nonlinear response of samples. Moreover, texture maps were drawn in order to deeply understand the effect of TG concentration on PPI structure during extrusion. This study elucidates the changes of protein structure at various stages of high-moisture extrusion and the influence mechanism of TG induction on protein extrusion structure, which is important for the optimization of extrusion conditions and the selection of appropriate materials to produce meat substitutes in the future.

## Materials and methods

### Materials

Pea protein isolate (PPI) was provided by Yantai Shuangta Food Co., Ltd., (Yantai) with a protein content of 84.2% (dry base). Moisture content was 7.3% (wet base). The other components were 6.5% (dry base) starch, 4.2% (dry base) ash, 2% (dry base) lipids, 3.1% (dry base) fiber. TG (120 U/g) was purchased from Jiangsu Dongsheng Biotechnology Co., Ltd. All other chemicals used were of analytical grade unless otherwise specified.

### Preparation of samples

Transglutaminase with PPI and distilled water were mixed in sealed bags. The concentration of TG was different in each group of samples (0, 0.25, 0.5, 1, 1.5, and 2%). The mixed samples were hydrated at 25°C for 30 min.

### Rheological properties

The rheological properties of PPI under various conditions were measured using a closed cavity rheometer (CCR) (RPA Elite, TA Instruments, New Castle, DE, United States). CCR measured the torque applied to the rheometer cavity by setting the lower chamber of the rheometer to oscillate at a specific frequency and strain amplitude. The upper chamber of the rheometer remained static, and grooves in the conical chamber prevented the specimen from sliding.

**FIGURE 1:**
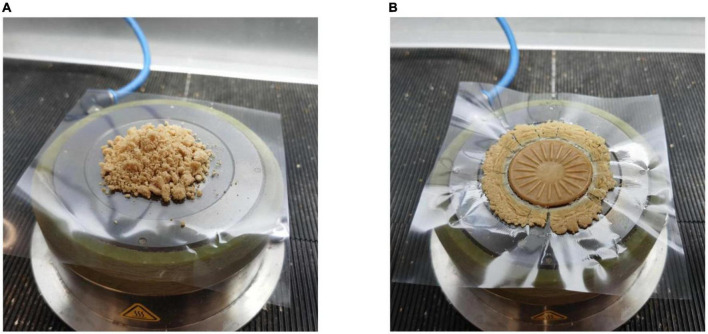
50 wt% PPI before **(A)** and after **(B)** measurement in the closed cavity rheometer.

In total, 5 g of hydrated samples were wrapped with two layers of plastic film and placed in the chamber of a closed chamber rheometer (Figure 1). The sealed samples were subjected to a pressure of about 4.5 bar (15, 18, 23). In order to simulate the state of protein samples at various extrusion temperatures, three different temperature conditions were tested: (A) 30°C, (B) from 30 to 120°C at a rate of 2°C/min and (C) from 30 to 120°C at a rate of 2°C/min and then cooled to 30°C. The frequency of 1 Hz and strain amplitude of 1% were always maintained during heating. Strain sweep tests were carried out on protein samples under the above mentioned three temperature conditions (0.1–1,000%). In order to make the obtained data more intuitive, the data at the end of the linear viscoelastic (LVE) regime and the intersection point of elastic modulus and viscous modulus were drawn into texture maps, so as to better understand the effect of TG on PPI texture under extrusion conditions. Then the strain amplitude was kept constant at the above mentioned three temperature conditions, and frequency scanning was carried out (the frequency varied from 0.1 to 50 Hz).

The data obtained from the large amplitude oscillatory shear test were analyzed. Lissajous curves were used to describe the relationship between the sample and the strain applied. The complexity of Lissajous curve shape depends on the system response to sinusoidal deformation. The presence of many harmonic components in these responses leads to the diversity of the Fourier spectrum. We can obtain the internal microstructure information of the test sample indirectly through the Lissajous curve. This helps to understand the rheological behavior of protein samples at large strain amplitude in more detail, and then better predict the rheological properties of the sample in the processing.

The closed area in the Lissajous curve represents the energy consumed per unit volume over a full period of the strain applied (24). The energy dissipated per unit volume in a single cycle (1) is only the first-order viscous Fourier coefficient (G'_1_: calculated from the intensity and phase of the first harmonic);


(1) $$Ed=\oint \;\text{σ}d\gamma =G{\text{"}}_{1}\gamma_{0}^{2}$$

The energy dissipated by a perfectly plastic material in a single cycleis equal to


(2) $${\left(Ed\right)}_{pp}={\text{4γ}}_{0}{\text{σ}}_{\max}$$

for a given strain amplitude (γ_0_) and a maximum stress (σ max).

Data of LAOS obtained were further analyzed according to the energy dissipation ratio (ϕ) proposed by Ewoldt et al. (24).


(3) $$\text{ϕ}=\frac{\text{Ed}}{\left(E\text{d}\right)\text{pp}}=\frac{\pi G_{1}^{"}\gamma_{0}}{4\sigma \text{max}}$$

### Determination of protein solubility

In order to reveal the interactions during structure formation and maintenance, eight extraction solvents were prepared to measure the protein solubility of the samples and calculate the changes in chemical crosslinking bonds, as showed in Table 1 (9, 25, 26). The soluble protein content in the supernatant was determined by the BCA method at 562 nm with a 96-well plate, and the total protein content of the samples was determined by the Kjeldahl method, and then the protein solubility was calculated according to the ratio between the two. Different chemical bonds were calculated based on solubility in different solutions. Each measurement shall be made in triplicate.

**TABLE 1:** Information about the extracting solutions, and calculation of chemical bonds and their interactions.

No.	Extracting solution	Chemical bond and their interactions	Calculation of chemical bonds and their interactions
(1)	0.035 mol/L pH 7.6 phosphate buffer solution (P)	Native state protein	(1)
(2)	8 mol/L urea in the phosphate buffer solution (P + U)	Hydrogen bonds	(2) − (1)
(3)	0.1 mol/L 2-mercaptoethanol (2-ME) in the phosphate buffer solution (P + M)	Disulphide bonds	(3) − (1)
(4)	1.5 g/100 mL sodium dodecyl sulfate (SDS) in the phosphate buffer solution (P + S)	Hydrophobic interactions	(4) − (1)
(5)	8 mol/L urea and 0.1 mol/L 2-ME in the phosphate buffer solution (P + U + M)	Interactions between hydrogen bonds and disulphide bonds	(5) − (2) − (3) + (1)
(6)	1.5 g/100 mL SDS and 8 mol/L urea in the phosphate buffer solution (P + U + S)	Interactions between hydrogen bonds and hydrophobic interactions	(6) − (2) − (4) + (1)
(7)	1.5 g/100 mL SDS and 0.1 mol/L 2-ME in the phosphate buffer solution (P + S + M)	Interactions between disulphide bonds and hydrophobic bonds	(7) − (3) − (4) + (1)
(8)	8 mol/L urea, 1.5 g/100 mL SDS and 0.1 mol/L 2-ME in the phosphate buffer solution (P + U + S + M)	Interactions among hydrogen bonds, disulphide bonds and hydrophobic interactions	(8) + (2) + (3) + (4) −(1) − (5) − (6) − (7)

### Statistical analysis

All experiments were carried out in three replicates. Results were reported as mean ± standard deviation. SPSS was used for one-way analysis of variance. *P* < 0.05 indicated a significant difference among groups.

## Results and discussion

### Small amplitude oscillatory shear

**FIGURE 2:**
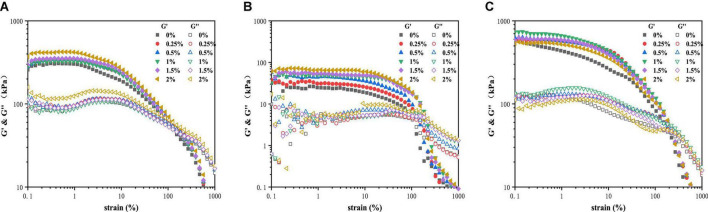
Strain sweep for PPI with different TG concentrations at **(A)** 30°C, **(B)** from 30 to 120°C and **(C)** from 30 to 120°C and then cooled to 30°C.

Figure 2 showed the storage (G') and loss (G”) modulus of PPI with TG (0–2%) varied with strain amplitude from 1 to 1,000% under the three temperature conditions. In the LVE regime, the modulus was independent of the strain amplitude applied. G' of the sample was always higher than G”, and the structure showed a solid state. The physical bond rupture rate increases faster than the reforming rate with the increase of strain amplitude. The decrease of G' by an order of magnitude and the fluidity of the sample was enhanced indicated that the structure of all PPI samples was destroyed (27). The value of G” was higher than G' indicates that the sample changed from a viscoelastic solid to a viscoelastic fluid (28).

As shown in Figure 2A, the G' of PPI treated by 2% TG in LVE regime was the maximum, about 450 KPa, and the control group (0% TG) had the lowest G', about 300 KPa. Figure 2B showed the curves of G' and G” for PPI when heated to 120 °C. The value of G' was higher than G” in LVE regime, indicating that PPI still remains elastic at this time (29, 30). However, compared with 30°C, the G' and G” of PPI decreased by an order of magnitude at 120°C. With the slow rise of temperature, TG could catalyze the cross-linking between PPI molecules and form a firm network structure (16). As a whole, G' showed a gradually increasing trend with the increase of TG concentration, and the G' of PPI with 2% TG was the largest, about 70 KPa. This was due to the catalytic action of TG to stabilize the conformation of protein molecules (31, 32). PPI was cooled to 30°C after heat treatment and its G' and G” changes were shown in Figure 2C. The modulus of the PPI was higher than 30°C after cooling. It was worth noting that after cooling to 30°C, the G' of PPI samples with 1% TG was the largest, about 750 KPa, which was larger than that of PPI samples with 1.5 and 2% TG. The reason might be that the addition of TG could promote the formation of new hydrogen and disulfide bonds to stabilize protein conformation in protein molecular rearrangement. However, excessive TG (2%) catalyze was not conducive to molecular rearrangement. The modulus was lower than that of other samples with TG after being cooled to 30°C (9).

**FIGURE 3:**
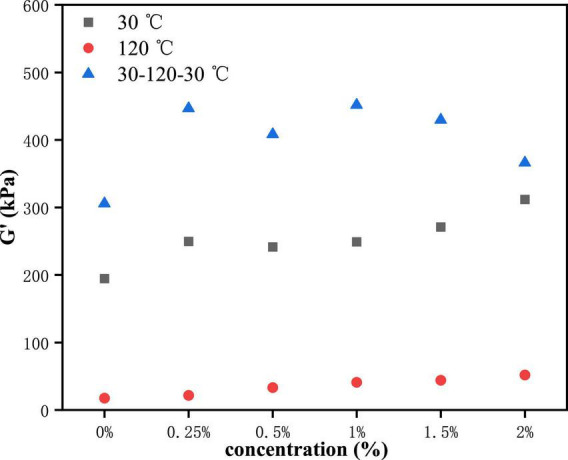
The function relation between the TG concentration and the value of G' at the end of the LVE regime.

The yield stress is commonly one of the symbols of the rheological properties of the sample. It is defined as the shear stress value at the end of the LVE regime. To make the test results more explicit, we researched the change in the yield stress of PPI with TG. Here, the end of the LVE regime is defined as the point where the difference in G' value is greater than 10%. As can be seen from Figure 3, G' showed an upward trend in general at the LVE regime. This indicated that the increase of TG concentration led to the enhancement of deformation resistance of samples (32, 33). At 120°C, the yield stress of the control group (0% TG) was less than that of the 30°C, which was caused by the high temperature leading to the protein structure fracture. However, the PPI samples showed different results after being cooled to 30°C. Compared with the sample at 30°C, the increase value of G' was the highest when the addition of TG was 1%, it meant that the effect of TG was the best at this concentration. This was due to the formation of new chemical bonds in the cooling process, which further increased the modulus of the PPI (9).

The G' and G” of PPI as a function of frequency were shown in Supplementary Figure 1. The G' and G” of PPI samples with different TG concentrations increased slightly with the increase of frequency (17). In order to show the difference between data more intuitively, the power law equation G' ∼ω*^n^* was used to fit the frequency response, where ω was frequency and n was exponent, and the significance analysis was conducted. As shown in Supplementary Table 1, the samples treated by different TG concentration had similar frequency dependence. At 120°C, the n of the samples was smaller than the initial, and the samples cooled after heating had the minimum n. The smaller n value in the power law equation indicates that the structure was more stable, and the change of this state required a stronger force. This weak power law behavior was similar to soft glass materials (34). PPI showed a disorder of metastable structure in this state.

### Texture maps

**FIGURE 4:**
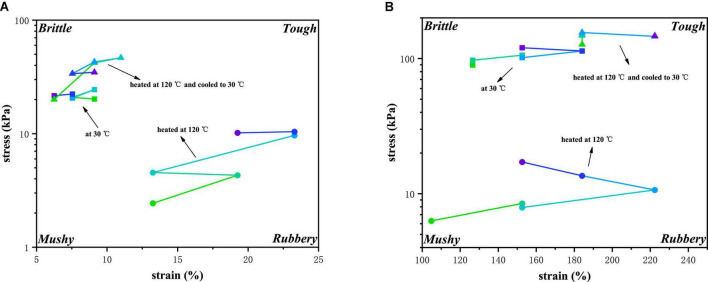
Texture map for the PPI with different TG concentrations **(A)** at the end of the LVE regime and **(B)** at the crossover point.

Although the texture of food can be perceived by the human senses, it can also be quantified into mechanical or rheological properties. These properties can be associated with “textures.” Texture map as a tool response can more intuitively show the differences between materials by reducing the nonlinear rheological information to a two-dimensional diagram only involving the key parameters of the characteristics (19). To make the texture maps representative, points at the end of the LVE regime and at the crossover point were selected for analysis (19). Figure 4 divided the response of samples into four quadrants-rubbery, mushy, brittle and tough. Table 2 showed various color schemes to describe points on the maps. The samples in the lower right corner are all “rubbery” and show low shear stress and high shear strain in rheological behavior. The representative materials are glutinous rice starch (35). The upper right quadrant represents a hard material with high shear stress and strain, such as fruit glue, with a “tough” texture. These materials, clustered in the upper left corner, have “brittle” properties, such as cheese (36). The materials in the lower left corner have low stress and low strain properties, and these materials exhibit a “mushy” texture, such as debranched starch (35).

**TABLE 2:** Color scheme to describe the points of the maps.

	0%TG	0.25%TG	0.5%TG	1%TG	1.5%TG	2%TG
At 30°C	■	■	■	■	■	■
Heated at 120°C	⏺	⏺	⏺	⏺	⏺	⏺
Heated at 120°C and cooled to 30°C	▲	▲	▲	▲	▲	▲

Figure 4A showed the texture map of PPI samples with different TG concentrations at three various temperatures (30°C, 120°C, and from 120°C cooled to 30°C). At 30°C, PPI samples were fragile, and with the increase of TG concentration, PPI samples were more brittle. After heating to 120°C, the PPI sample moved to the lower right of the texture map as a whole and became more rubbery than mushy. The higher concentration of TG resulted in higher stress values in the samples. After being cooled to 30°C, the PPI sample moved back to the upper left of the texture map, meaning that the sample became more brittle. This suggested that additional interactions were formed as the sample cooled. It could be seen from Figure 4A that the sample with 1% TG had the maximum stress value, and the sample with 1.5% TG was more brittle. It indicated that the concentration of TG was not correlated with the stress value, which was similar to Figure 2. These phenomena indicated that appropriate TG could promote the formation of new forces in the cooling process of the sample, while the effect of excessive TG is not satisfactory. The texture of PPI samples with different concentration of TG at the crossover points was shown in Figure 4B. At 30°C, the samples were brittle. However, we could see that the overall samples gradually toughed with the increase of TG addition. At 120°C, although all PPI samples were softened by heating, the strain values of the samples with TG were stronger than those of samples without TG, and showed a positive correlation trend. Under the condition of cooled to 30°C, the overall characteristics of the sample changed to brittle. It was much tougher than 30°C. These phenomena also confirmed the correctness of the data obtained before.

### Large amplitude oscillatory shear

**FIGURE 5:**
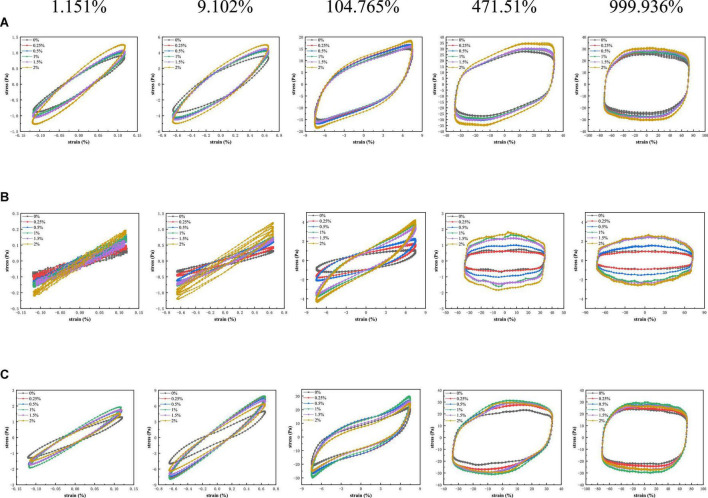
Lissajous curves of stress versus strain amplitude for PPI with different TG concentrations at **(A)** 30°C, **(B)** from 30 to 120°C and **(C)** from 30 to 120°C and then cooled to 30°C.

The LAOS provided a great deal of additional information about the microstructure of complex fluids (37–39). Figure 5 showed Lissajous curves of PPI samples with different TG concentrations under different strain amplitudes at three various temperatures. The Lissajous curves were similar at the three temperature conditions (30°C, 120°C, and from 120°C cooled to 30°C).

At 30°C, the change of strain amplitude almost did not affect the performance of PPI samples treated by different concentration of TG. It was observed that the area of the graph showed a positive correlation with the strain amplitude. PPI with different TG concentrations showed a narrow oval shape with a small closed area at the strain amplitude of 1.151%, which means that the specimen has elastic response (38). As the strain increases, the graph gradually approximates to a rectangle, which is a sign of viscous dissipation. This indicated that the system dissipated more and more mechanical energy in a single cycle. This showed that the internal structure of the sample had changed due to the action of external forces and the storage modulus decreased, and finally showed the plastic (40–42). As can be seen from Figure 5, PPI with 2% TG reduced the energy dissipation in the deformation process. At 120°C, the addition of TG affected the shape of Lissajous curve, presenting an inverted S-shaped curve at a high strain amplitude (104.765%). The stress value of PPI with TG was higher than that of the control group (0% TG). It was positively correlated with the additional concentration of TG at various strain amplitudes. This indicated that PPI was aggregated treated by TG and protein conformation was stabilized at 120°C. When the sample cooled to 30°C, the stress value of PPI with TG was greater than that of the control group (0% TG). The stress value of PPI with 1% TG was the highest. The reason may be that the addition of TG promotes the formation of new chemical bonds during the rearrangement of protein molecules, which makes the protein structure more stable. However, excessive addition of TG was not conducive to molecular rearrangement, resulting in lower stress values than PPI with 1% TG after being cooled to 30°C (9).

### The energy dissipation ratio

**FIGURE 6:**
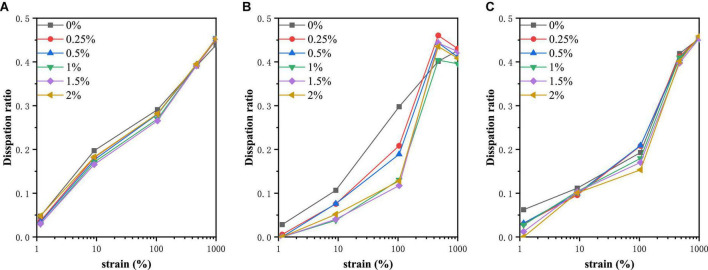
Dissipation ratio of PPI with different TG concentrations at **(A)** 30°C, **(B)** from 30 to 120°C and **(C)** from 30 to 120°C and then cooled to 30°C.

In order to make the energy dissipation and nonlinear behavior in the process more clearly, we calculate the dissipation ratio ϕ. The function of energy dissipation ratio changing with strain value was shown in Figure 6. The ϕ was small for PPI at small strain amplitudes, showing a predominantly elastic response. There was a positive correlation between dissipation ratio and strain amplitude. As can be seen from Figure 6, the maximum dissipation ratio obtained was about 0.45, showing a viscous characteristic. At 30°C, the energy dissipation ratio of each PPI sample had no significant difference. However, at 120°C, PPI without TG had the largest dissipation ratio with the small strain amplitude, which was manifested as viscosity. When the strain reached 104.765%, the dissipation ratio of the PPI with TG was suddenly increased, and finally tended to be the same. And the overall performance was that PPI samples with higher concentration of TG had high elasticity. PPI with different TG concentrations were slightly different in general when cooled to 30°C. At a small strain amplitude, the elasticity of the sample was positively correlated with the addition of TG. When the strain amplitude reached 1,000%, the energy dissipation ratio of different samples tended to 0.45. The additional information about the dissipation behavior verifies the correctness of the changes in the closed regime of the Lissajous curve. This helps to understand the rheological behavior of PPI in a directive way.

### Interactive forces involved in the formation and maintenance of the structure

Based on the solubility of PPI, the proportion of different chemical bonds and their interactions were calculated, as showed in Table 3. The results showed that the trend of the proportion of chemical bonds was slightly different before and after heat treatments. Compred with unheated sample, the number of disulfide bonds in heated samples significantly increased, suggesting that heating promoted the formation of new disulfide bonds. Table 3 showed that compared with the control group (0% TG), the number of hydrogen bonds was significantly increased and the hydrophobic interaction was enhanced to maintain the protein structure in the samples with TG at 30°C. While the number of disulfide bonds decreased, the interaction between hydrogen bond and hydrophobic interaction did not change significantly. Therefore, we could obtain the information that TG promotes the unfolding of protein molecular chain by breaking disulfide bonds and electrostatic forces within the molecule (6). After heat treatment, the number of hydrogen bonds and hydrophobic effect of the sample increased significantly with the increase of TG concentration. On the other hand, the number of disulfide bonds increased at a certain concentration of TG (less than 1.5%), the number of disulfide bonds decreased with the further increase of TG concentrations, but it was still larger than the control group (0% TG). This phenomenon was also reflected in the complex interactions between various chemical bonds. In conclusion, excessive modification of TG is not conducive to the formation of disulfide bonds, but the disulfide bond is still the main force to maintain the structure of PPI.

**TABLE 3:** Effects of TG on chemical cross-linking of PPI at 30°C and from 30 to 120°C and then cooled to 30°C.

Temperature	TG	P	P+U	P+M	P+S	P+U+M	P+U+S	P+S+M	P+U+S+M
30°C	0%	0.53 ± 0.02^b^	5.09 ± 0.55^ab^	44.30 ± 0.99^a^	4.26 ± 0.72^bc^	−14.63 ± 1.50b	−4.90 ± 0.28a	−4.12 ± 4.40a	5.85 ± 6.69^a^
	0.25%	0.71 ± 0.82^b^	4.49 ± 1.60^b^	44.35 ± 0.66^a^	3.84 ± 1.52^c^	−7.70 ± 2.43a	−2.63 ± 2.39a	−11.00 ± 1.14b	7.84 ± 3.82^a^
	0.5%	0.44 ± 0.07^b^	4.54 ± 0.76^b^	42.15 ± 2.21^a^	4.65 ± 1.05^abc^	−8.02 ± 1.09a	−3.34 ± 2.08a	−1.89 ± 3.35a	1.31 ± 2.54^a^
	1%	0.26 ± 0.22^b^	6.27 ± 1.44^ab^	41.70 ± 2.00^a^	5.89 ± 2.00^abc^	−11.32 ± 2.32*ab*	−4.23 ± 1.51a	−6.03 ± 2.15*ab*	5.81 ± 1.80^a^
	1.5%	1.92 ± 0.34^a^	7.02 ± 1.04^a^	36.92 ± 1.02^b^	6.55 ± 0.02^ab^	−7.58 ± 2.78a	−5.48 ± 1.16a	−6.04 ± 2.15*ab*	6.01 ± 1.80^a^
	2%	1.21 ± 0.86^ab^	6.24 ± 1.06^ab^	37.73 ± 1.54^b^	6.82 ± 0.90^a^	−8.09 ± 2.00a	−5.04 ± 1.31a	−6.71 ± 2.44*ab*	2.59 ± 1.17^a^
Cooled to 30°C	0%	0.48 ± 0.04^ab^	2.65 ± 1.59^ab^	64.97 ± 4.60^ab^	0.73 ± 0.85^c^	−21.33 ± 1.70b	1.15 ± 1.07^a^	−12.15 ± 1.19a	7.11 ± 8.64^a^
	0.25%	0.34 ± 0.02^b^	1.31 ± 0.46^b^	55.50 ± 18.08^b^	3.23 ± 0.15^ab^	−22.51 ± 18.79a	−0.86 ± 0.62*ab*	−12.50 ± 14.65a	6.83 ± 12.25^b^
	0.5%	0.82 ± 0.44^a^	1.77 ± 1.49^ab^	68.49 ± 3.46^ab^	0.78 ± 0.78^c^	−22.97 ± 4.04b	1.31 ± 1.95^a^	−13.71 ± 3.42a	5.05 ± 3.03^a^
	1%	0.57 ± 0.06^ab^	2.35 ± 1.80^ab^	67.95 ± 6.75^ab^	2.09 ± 1.77^bc^	−26.74 ± 6.79b	−0.39 ± 1.16*ab*	−14.37 ± 8.73a	15.39 ± 13.99^a^
	1.5%	0.52 ± 0.07^ab^	4.36 ± 0.06^a^	74.76 ± 2.72^a^	4.66 ± 0.00^a^	−26.48 ± 1.19b	−3.44 ± 0.40c	−14.68 ± 4.44a	6.78 ± 1.71^a^
	2%	0.68 ± 0.19^ab^	2.99 ± 1.10^ab^	60.40 ± 2.84^ab^	3.16 ± 0.70^ab^	−16.83 ± 1.08b	−2.01 ± 0.83*bc*	−0.65 ± 1.73a	−0.62 ± 2.17*ab*

Values are expressed as the means and standard deviations of three measurements. Different letters in the same line indicate significant differences between groups (*P* < 0.05).

## Conclusion

In this study, we simulated the processing conditions of plant-based meat products using CCR to elucidate the effect of TG crosslinking on PPI structure changes during high-moisture extrusion. The results showed that G' of PPI showed a rising trend with the increase of TG concentration at 120°C. Compared with the control group (0% TG), PPI with TG was significantly harder in texture. After being cooled to 30°C, the G' of PPI with 1% TG was the highest. In conclusion, the addition of TG could enhance the texture of PPI, but excessive TG (2%) had the opposite effect. Overall, this study provides an effective characterization method for elucidating the structural changes of extruded materials during high-moisture extrusion. It is helpful for us to further explore the mechanism of protein extrusion. These findings can provide guidance for the production and processing of plant-based meat products.

## Data availability statement

The original contributions presented in this study are included in the article/Supplementary material, further inquiries can be directed to the corresponding authors.

## Author contributions

JQ and YZ were involved in the protocol design, data analyses, and in the interpretation of results and drafted the manuscript. XL and JZ were involved in conceptualization and funding acquisition. JL and GZ conceived and designed this study. All authors proofread and approved the final manuscript.

## References

[1] Cutroneo S Angelino D Tedeschi T Pellegrini N Martini D Group SYW. Nutritional quality of meat analogues: results from the food labelling of Italian products (FLIP) project. *Front Nutr.* (2022) 9:852831. 10.3389/fnut.2022.852831 35558740 PMC9090485

[2] Peng H Zhang J Wang S Qi M Yue M Zhang S High moisture extrusion of pea protein: effect of l-cysteine on product properties and the process forming a fibrous structure. *Food Hydrocoll.* (2022) 129:107633. 10.1016/j.foodhyd.2022.107633

[3] He J Evans NM Liu HZ Shao SQ. A review of research on plant-based meat alternatives: driving forces, history, manufacturing, and consumer attitudes. *Compr Rev Food Sci Food Saf.* (2020) 19:2639–56. 10.1111/1541-4337.12610 33336979

[4] Dekkers BL Boom RM van der Goot AJ. Structuring processes for meat analogues. *Trends Food Sci Tech.* (2018) 81:25–36. 10.1016/j.tifs.2018.08.011

[5] Kyriakopoulou K Keppler JK van der Goot AJ. Functionality of ingredients and additives in plant-based meat analogues. *Foods.* (2021) 10:600. 10.3390/foods10030600 33809143 PMC7999387

[6] Gaspar AL de Goes-Favoni SP. Action of microbial transglutaminase (MTGase) in the modification of food proteins: a review. *Food Chem.* (2015) 171:315–22. 10.1016/j.foodchem.2014.09.019 25308675

[7] Fatima SW Khare SK. Current insight and futuristic vistas of microbial transglutaminase in nutraceutical industry. *Microbiol Res.* (2018) 215:7–14. 10.1016/j.micres.2018.06.001 30172311

[8] Yang X Zhang Y. Expression of recombinant transglutaminase gene in *Pichia pastoris* and its uses in restructured meat products. *Food Chem.* (2019) 291:245–52. 10.1016/j.foodchem.2019.04.015 31006466

[9] Zhang J Chen Q Liu L Zhang Y He N Wang Q. High-moisture extrusion process of transglutaminase-modified peanut protein: effect of transglutaminase on the mechanics of the process forming a fibrous structure. *Food Hydrocoll.* (2021) 112:106346. 10.1016/j.foodhyd.2020.106346

[10] Fu Y Chen T Chen F. The potentials and challenges of using microalgae as an ingredient toproduce meat analogues. *Trends Food Sci Tech.* (2021) 112:188–200. 10.1016/j.tifs.2021.03.050

[11] Emin MA Teumer T Schmitt W Rädle M Schuchmann HP. Measurement of the true melt temperature in a twin-screw extrusion processing of starch based matrices via infrared sensor. *J Food Eng.* (2016) 170:119–24. 10.1016/j.jfoodeng.2015.09.018

[12] Dou W Zhang X Zhao Y Zhang Y Jiang L Sui X. High moisture extrusion cooking on soy proteins: importance influence of gums on promoting the fiber formation. *Food Res Int.* (2022) 156:111189. 10.1016/j.foodres.2022.111189 35651098

[13] Chen Q Zhang J Zhang Y Kaplan DL Wang Q. Protein-amylose/amylopectin molecular interactions during high-moisture extruded texturization toward plant-based meat substitutes applications. *Food Hydrocoll.* (2022) 127:107559. 10.1016/j.foodhyd.2022.107559

[14] Chen FL Wei YM Zhang B. Chemical cross-linking and molecular aggregation of soybean protein during extrusion cooking at low and high moisture content. *LWT Food Sci Technol.* (2011) 44:957–62. 10.1016/j.lwt.2010.12.008

[15] Koch L Hummel L Schuchmann HP Emin MA. Influence of defined shear rates on structural changes and functional properties of highly concentrated whey protein isolate-citrus pectin blends at elevated temperatures. *Food Biophys.* (2017) 12:309–22. 10.1007/s11483-017-9487-2

[16] Koch L Emin MA Schuchmann HP. Reaction behaviour of highly concentrated whey protein isolate under defined heat treatments. *Int Dairy J.* (2017) 71:114–21. 10.1016/j.idairyj.2017.03.013

[17] Dekkers BL Boom RM van der Goot AJ. Viscoelastic properties of soy protein isolate - pectin blends: richer than those of a simple composite material. *Food Res Int.* (2018) 107:281–8. 10.1016/j.foodres.2018.02.037 29580487

[18] Wittek P Zeiler N Karbstein HP Emin MA. Analysis of the complex rheological properties of highly concentrated proteins with a closed cavity rheometer. *Appl Rheol.* (2020) 30:64–76. 10.1515/arh-2020-0107

[19] Schreuders FKG Sagis LMC Bodnár I Erni P Boom RM van der Goot AJ. Mapping the texture of plant protein blends for meat analogues. *Food Hydrocoll.* (2021) 118:106753. 10.1016/j.foodhyd.2021.106753

[20] Schreuders FKG Sagis LMC Bodnár I Erni P Boom RM van der Goot AJ. Small and large oscillatory shear properties of concentrated proteins. *Food Hydrocoll.* (2021) 110:106172. 10.1016/j.foodhyd.2020.106172

[21] Lu ZX He JF Zhang YC Bing DJ. Composition, physicochemical properties of pea protein and its application in functional foods. *Crit Rev Food Sci Nutr.* (2020) 60:2593–605. 10.1080/10408398.2019.1651248 31429319

[22] Lam ACY Can Karaca A Tyler RT Nickerson MT. Pea protein isolates: structure, extraction, and functionality. *Food Rev Int.* (2016) 34:126–47. 10.1080/87559129.2016.1242135

[23] Pietsch VL Bühler JM Karbstein HP Emin MA. High moisture extrusion of soy protein concentrate: influence of thermomechanical treatment on protein-protein interactions and rheological properties. *J Food Eng.* (2019) 251:11–8. 10.1016/j.jfoodeng.2019.01.001

[24] Ewoldt RH Winter P Maxey J McKinley GH. Large amplitude oscillatory shear of pseudoplastic and elastoviscoplastic materials. *Rheol Acta.* (2009) 49:191–212. 10.1007/s00397-009-0403-7

[25] Zhang J Liu L Jiang Y Shah F Xu Y Wang Q. High-moisture extrusion of peanut protein-/carrageenan/sodium alginate/wheat starch mixtures: effect of different exogenous polysaccharides on the process forming a fibrous structure. *Food Hydrocoll.* (2020) 99:105311. 10.1016/j.foodhyd.2019.105311

[26] Lin QQ Pan LB Deng NH Sang ML Cai KT Chen CL Protein digestibility of textured-wheat-protein (TWP) -based meat analogues: (I) Effects of fibrous structure. *Food Hydrocoll.* (2022) 130:107694. 10.1016/j.foodhyd.2022.107694

[27] Laukkanen O-V. Small-diameter parallel plate rheometry: a simple technique for measuring rheological properties of glass-forming liquids in shear. *Rheol Acta.* (2017) 56:661–71. 10.1007/s00397-017-1020-5

[28] Anvari M Joyner HS. Effect of formulation on structure-function relationships of concentrated emulsions: rheological, tribological, and microstructural characterization. *Food Hydrocoll.* (2017) 72:11–26. 10.1016/j.foodhyd.2017.04.034

[29] Coblas D Broboana D Balan C. Correlation between large amplitude oscillatory shear (LAOS) and steady shear of soft solids at the onset of the fluid rheological behavior. *Polymer.* (2016) 104:215–26. 10.1016/j.polymer.2016.06.003

[30] Khandavalli S Rothstein JP. Large amplitude oscillatory shear rheology of three different shear-thickening particle dispersions. *Rheol Acta.* (2015) 54:601–18. 10.1007/s00397-015-0855-x

[31] Wang K Luo S Cai J Sun Q Zhao Y Zhong XY Effects of partial hydrolysis and subsequent cross-linking on wheat gluten physicochemical properties and structure. *Food Chem.* (2016) 197:168–74. 10.1016/j.foodchem.2015.10.123 26616937

[32] Pöri P Nisov A Nordlund E. Enzymatic modification of oat protein concentrate with trans- and protein-glutaminase for increased fibrous structure formation during high-moisture extrusion processing. *LWT Food Sci Technol.* (2022) 156:113035. 10.1016/j.lwt.2021.113035 35650994

[33] Feng XL Liu HZ Shi AM Liu L Wang Q Adhikari B. Effects of transglutaminase catalyzed crosslinking on physicochemical characteristics of arachin and conarachin-rich peanut protein fractions. *Food Res Int.* (2014) 62:84–90. 10.1016/j.foodres.2014.02.022

[34] Bandyopadhyay R Liang D Harden JL Leheny RL. Slow dynamics, aging, and glassy rheology in soft and living matter. *Solid State Commun.* (2006) 139:589–98. 10.1016/j.ssc.2006.06.023

[35] Precha-Atsawanan S Uttapap D Sagis LMC. Linear and nonlinear rheological behavior of native and debranched waxy rice starch gels. *Food Hydrocoll.* (2018) 85:1–9. 10.1016/j.foodhyd.2018.06.050

[36] Tunick MH Van Hekken DL. Rheology and texture of commercial queso fresco cheeses made from raw and pasteurized milk. *J Food Qual.* (2010) 33:204–15. 10.1111/j.1745-4557.2010.00331.x

[37] Alghooneh A Razavi SMA Kasapis S. Classification of hydrocolloids based on small amplitude oscillatory shear, large amplitude oscillatory shear, and textural properties. *J Texture Stud.* (2019) 50:520–38. 10.1111/jtxs.12459 31226217

[38] Bozorgi Y Underhill PT. Large-amplitude oscillatory shear rheology of dilute active suspensions. *Rheol Acta.* (2014) 53:899–909. 10.1007/s00397-014-0806-y

[39] Mermet-Guyennet MRB Gianfelice de Castro J Habibi M Martzel N Denn MM Bonn D. LAOS: the strain softening/strain hardening paradox. *J Rheol.* (2015) 59:21–32. 10.1122/1.4902000

[40] Xia W Siu WK Sagis LMC. Linear and non-linear rheology of heat-set soy protein gels: effects of selective proteolysis of β-conglycinin and glycinin. *Food Hydrocoll.* (2021) 120:106962. 10.1016/j.foodhyd.2021.106962

[41] Huang ZG Wang XY Zhang JY Liu Y Zhou T Chi SY Effect of heat treatment on the nonlinear rheological properties of acid-induced soy protein isolate gels modified by high-pressure homogenization. *LWT Food Sci Technol.* (2022) 157:113094. 10.1016/j.lwt.2022.113094

[42] Patole S Cheng L Yang Z. Impact of incorporations of various polysaccharides on rheological and microstructural characteristics of heat-induced quinoa protein isolate gels. *Food Biophys.* (2022) 1–10. 10.1007/s11483-022-09720-335645654

