# Supplementary material for: Rheological properties of transglutaminase-treated concentrated pea protein under conditions relevant to high-moisture extrusion processing
Source: Front Nutr. 2022 Aug 12;9:970010. doi: 10.3389/fnut.2022.970010 (PMC9412734; doi:10.3389/fnut.2022.970010)
Supplement: Supplementary file 1 [file Data_Sheet_1.docx]

**Supplementary material**

**Fig. S1.** The storage and loss modulus of 50 wt% PPI as function of frequency (at 1% strain amplitude) at three temperature conditions.

30 °C 120 °C 30-120-30 °C


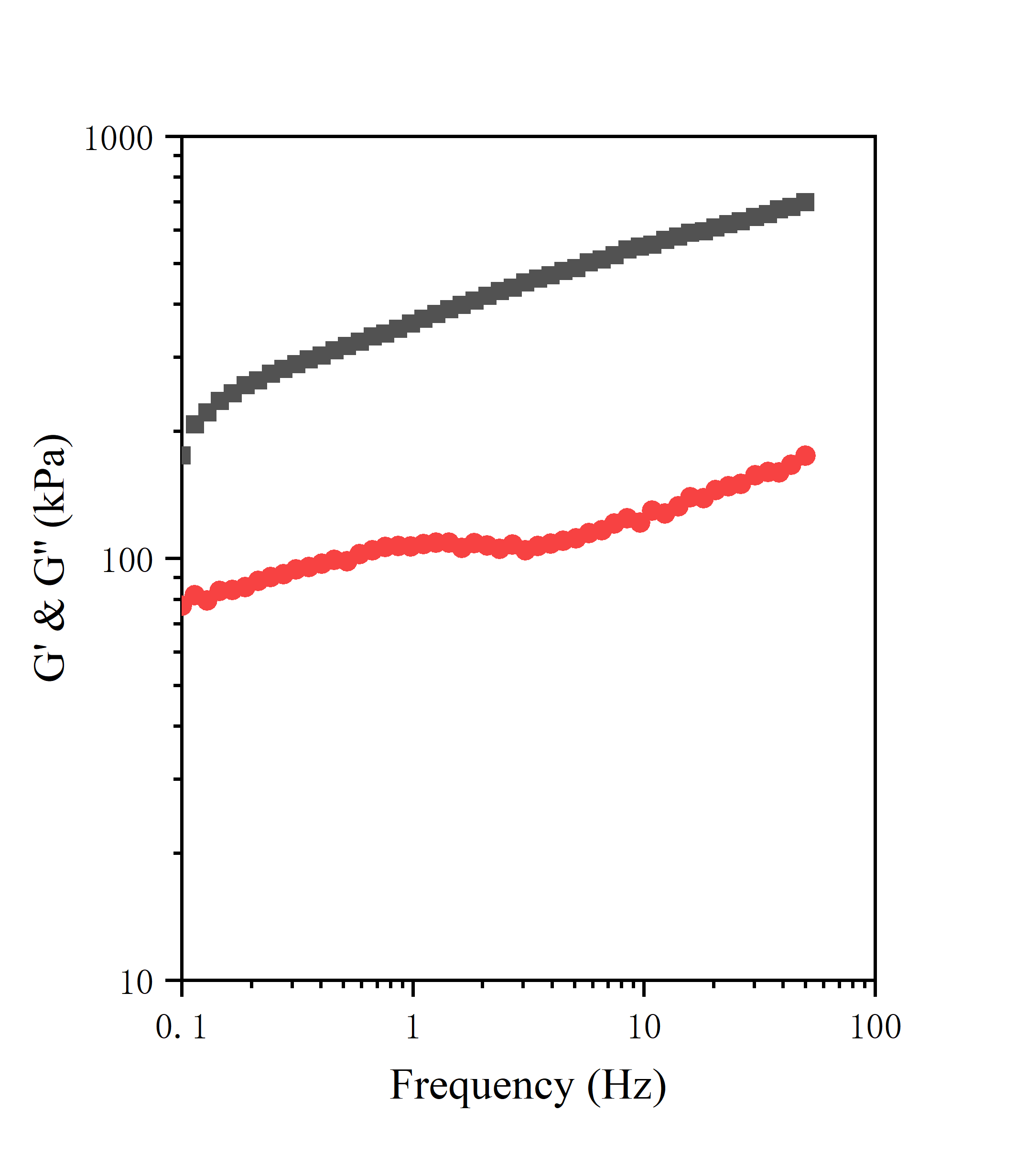

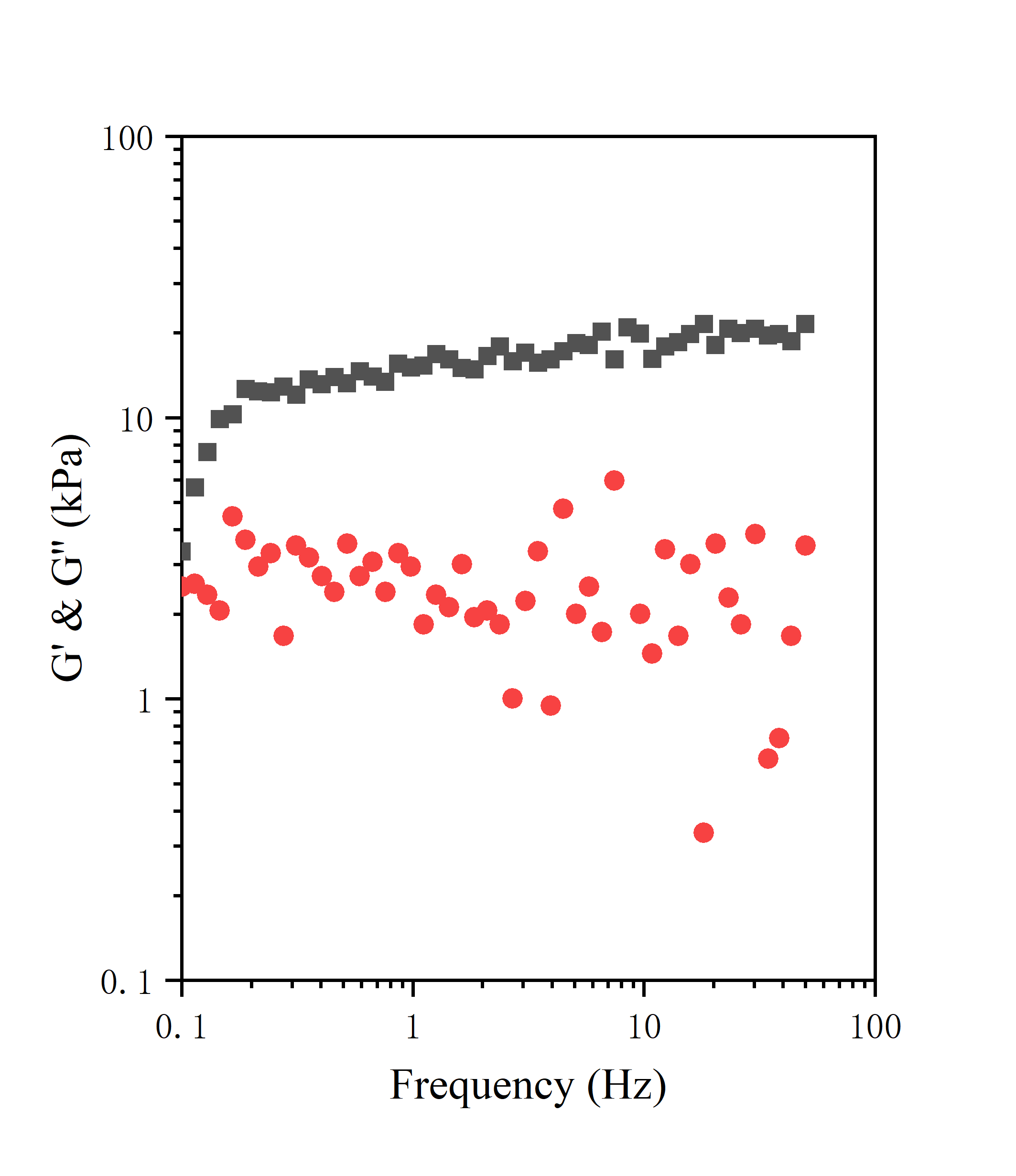

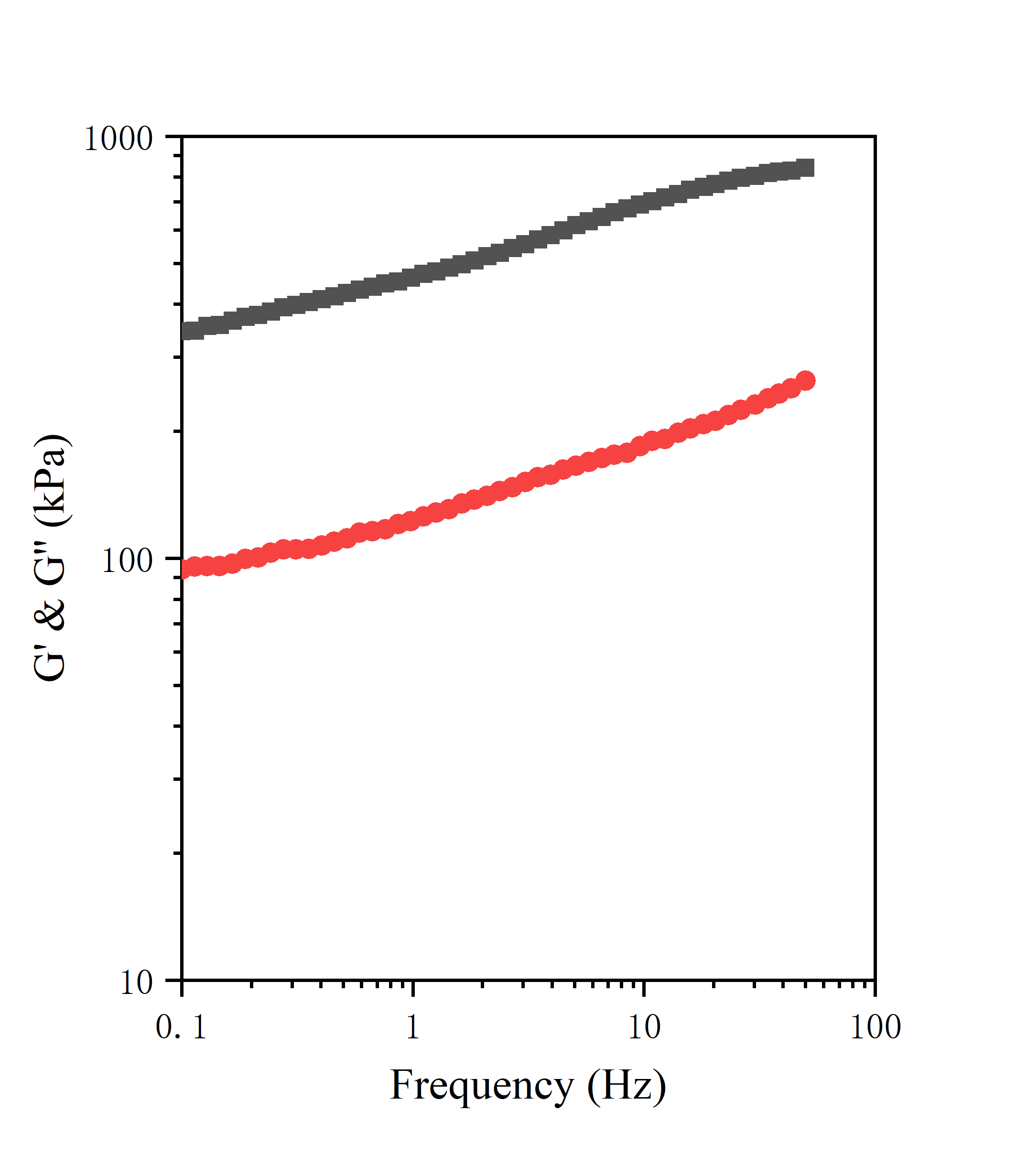


0%TG


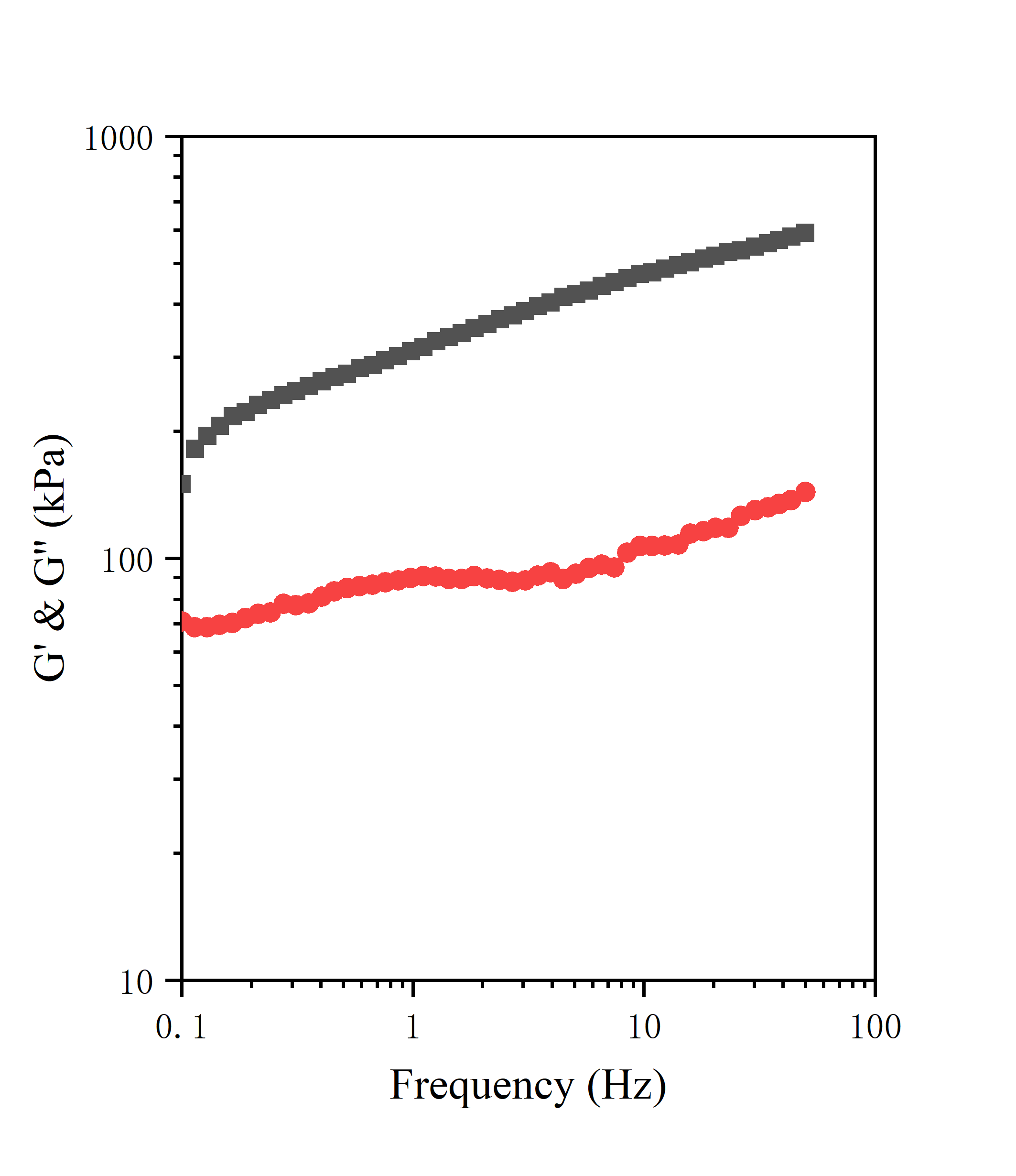

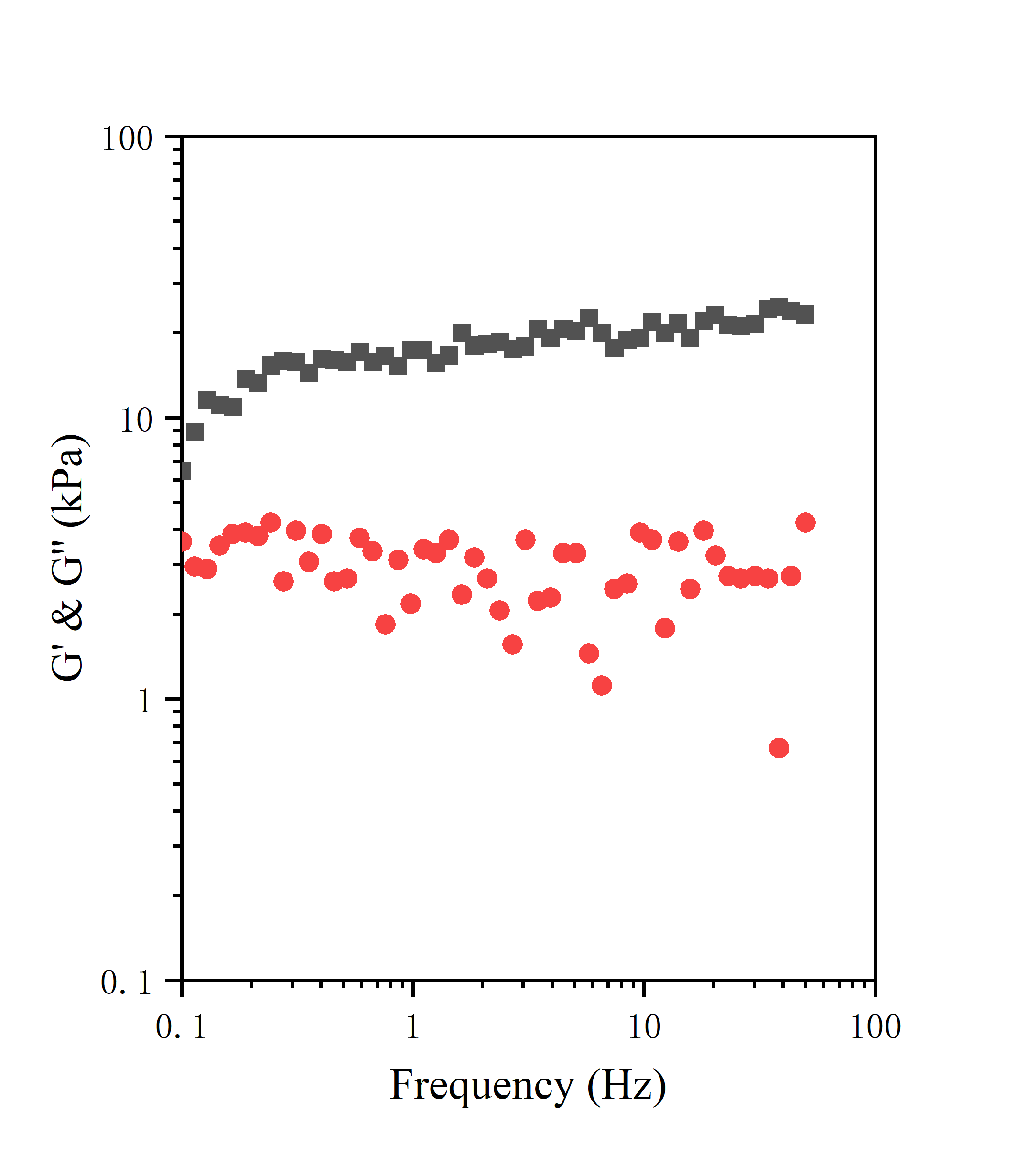

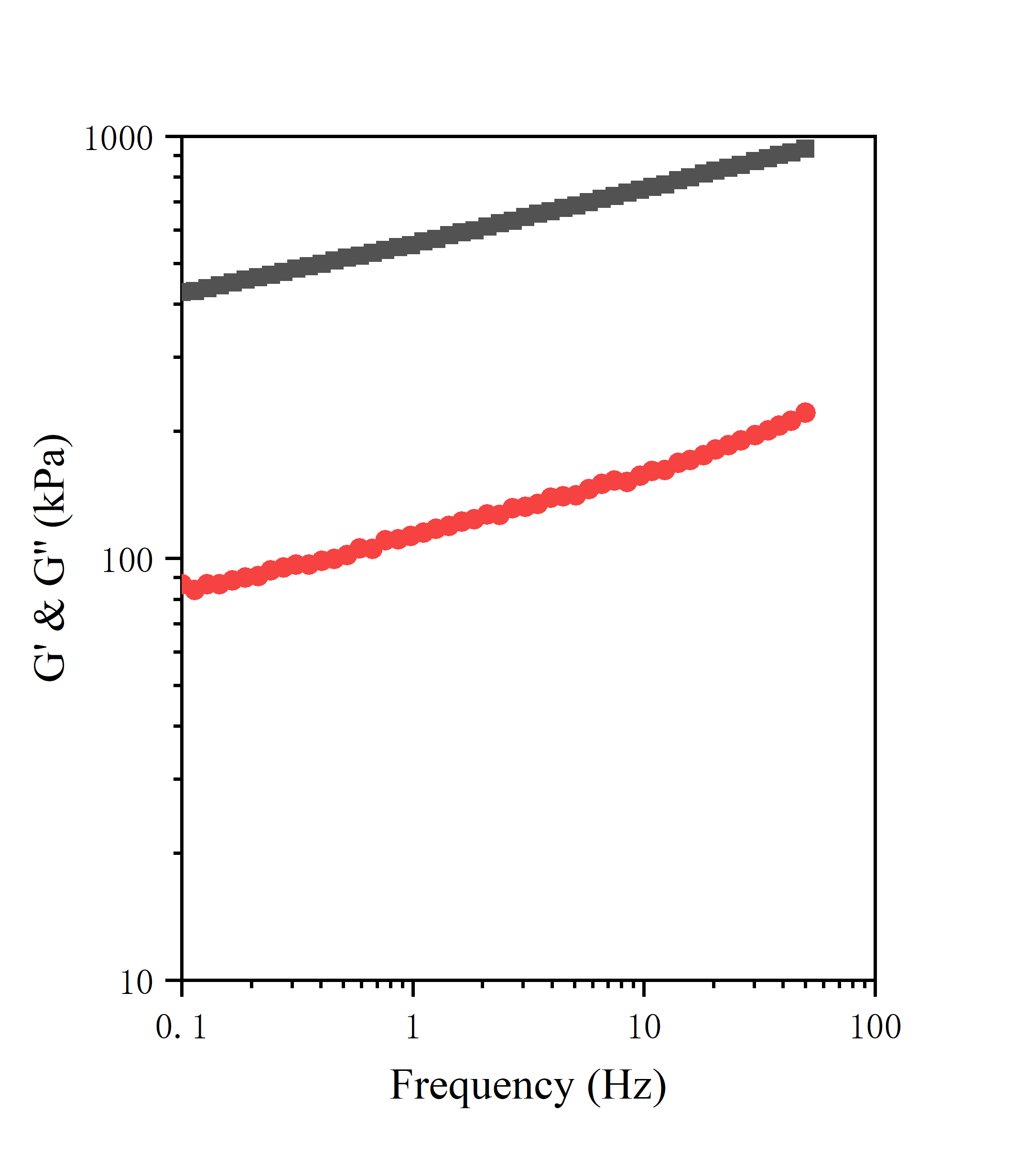


0.25%TG


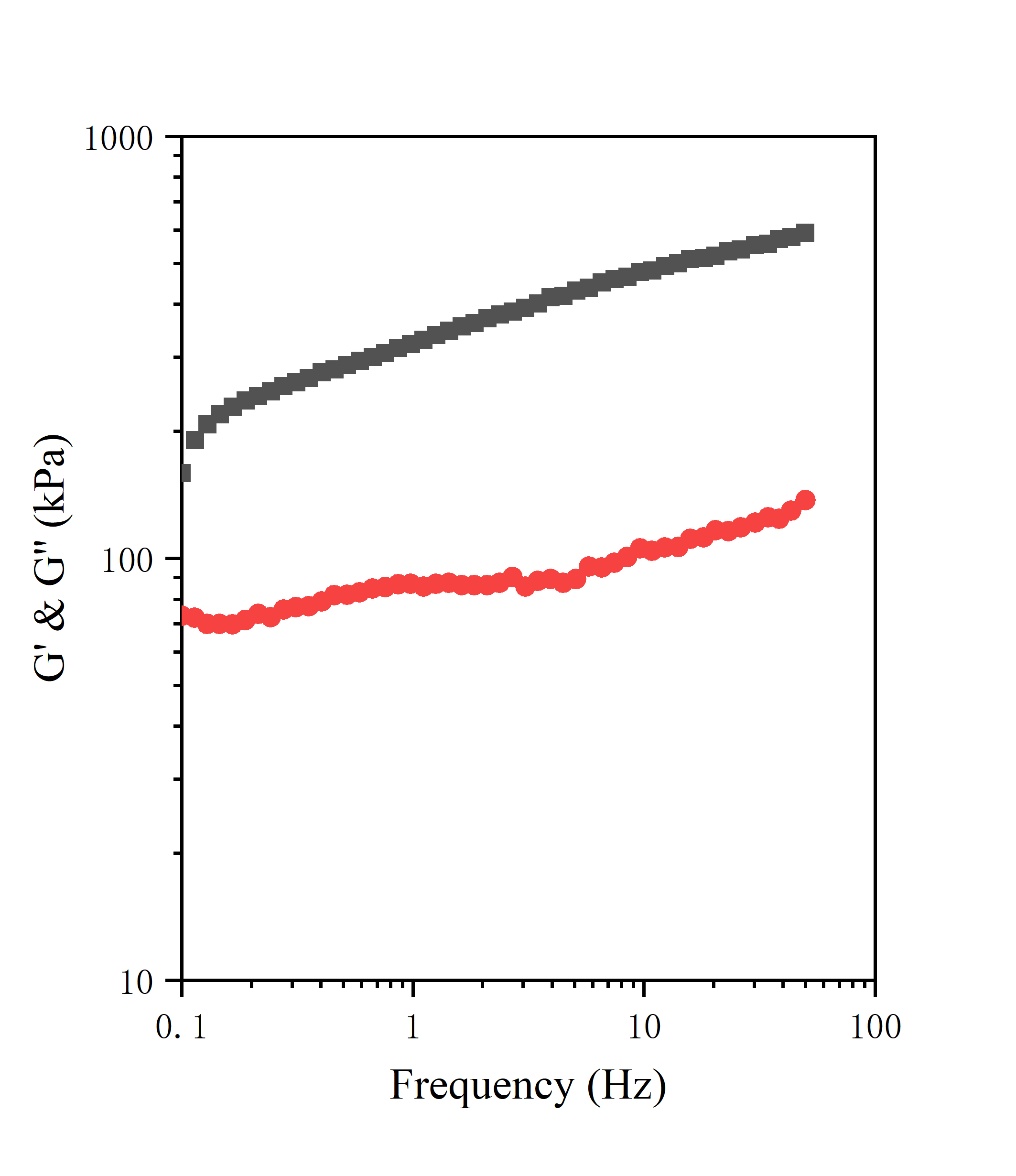

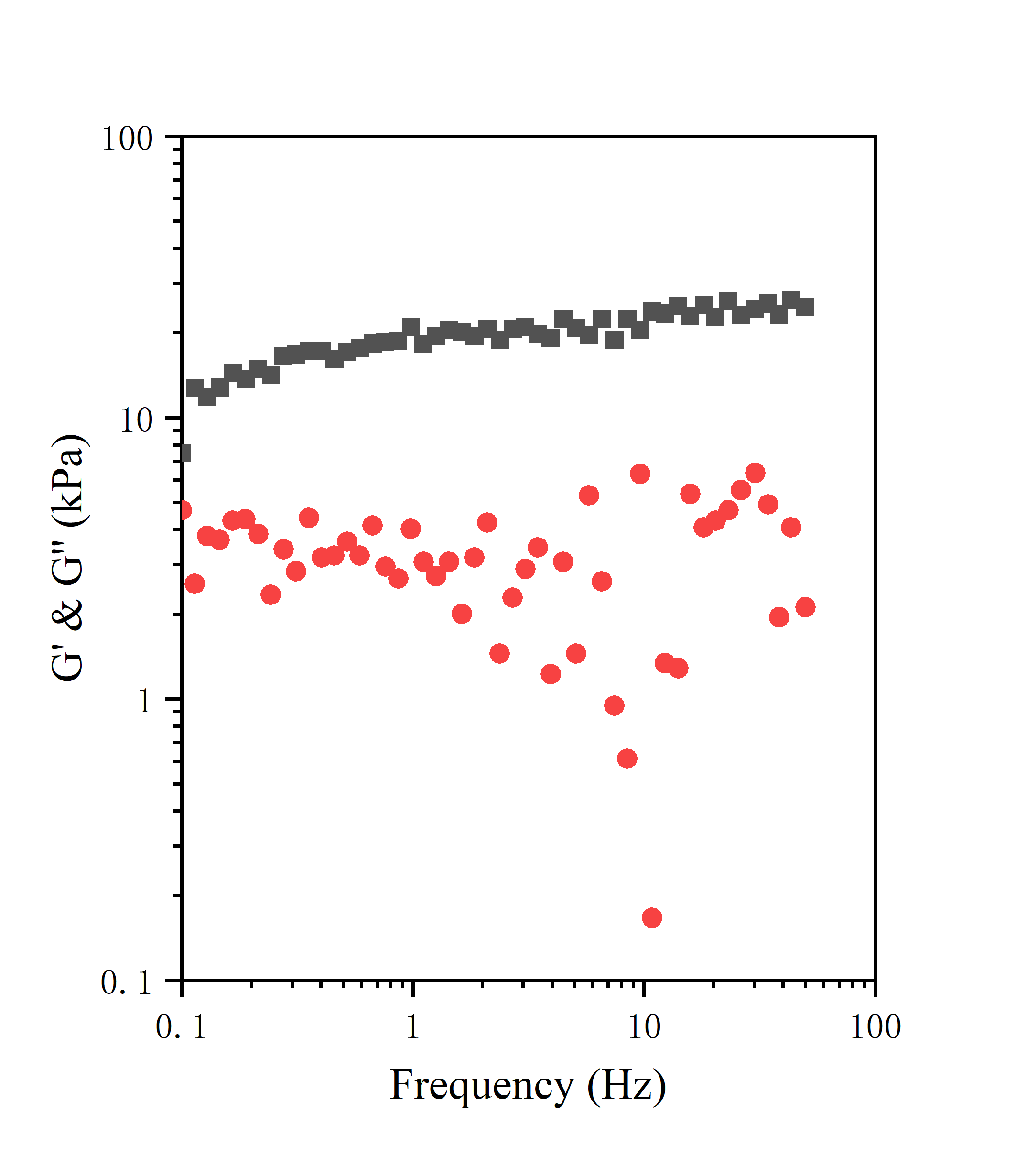

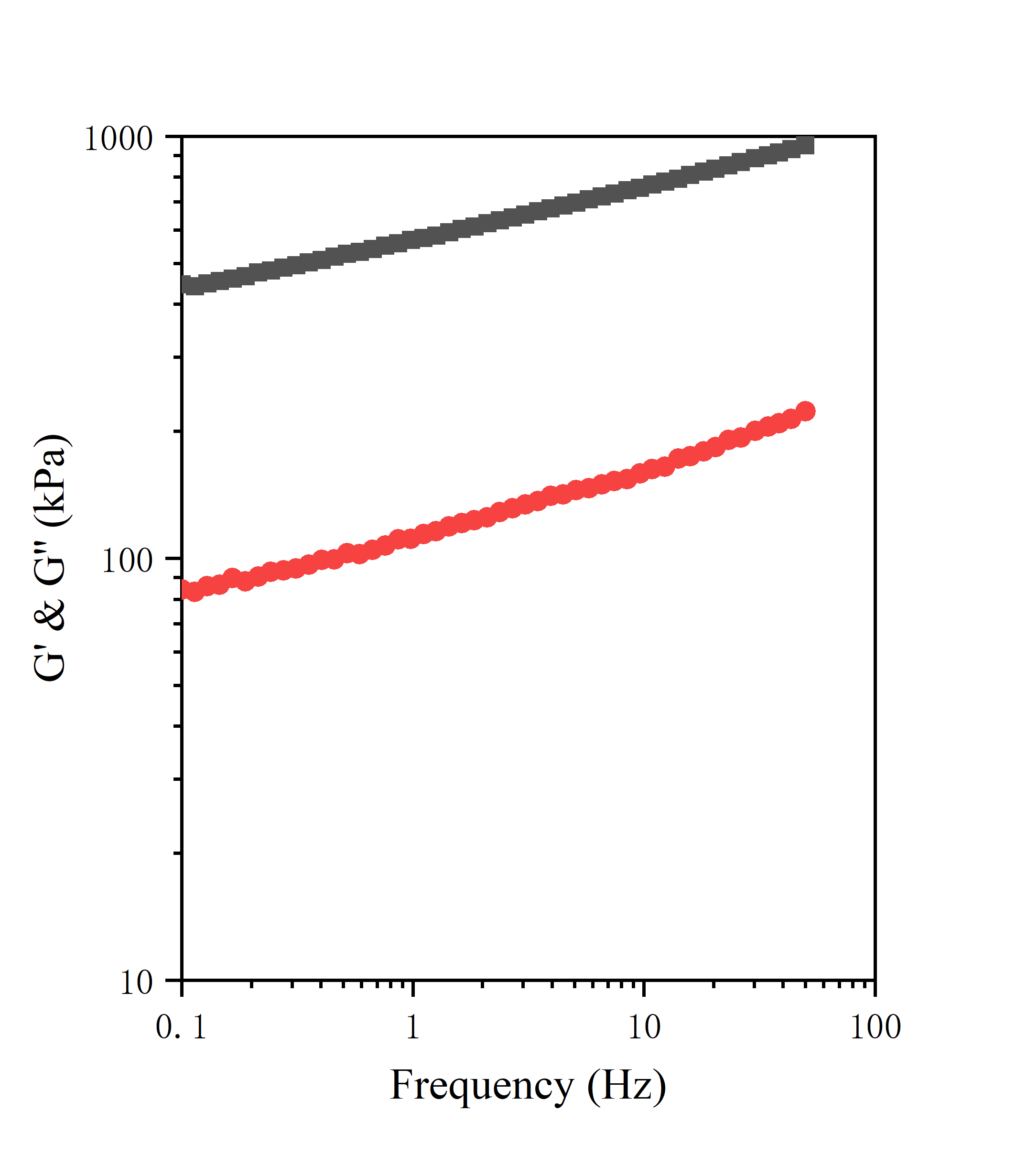


0.5%TG


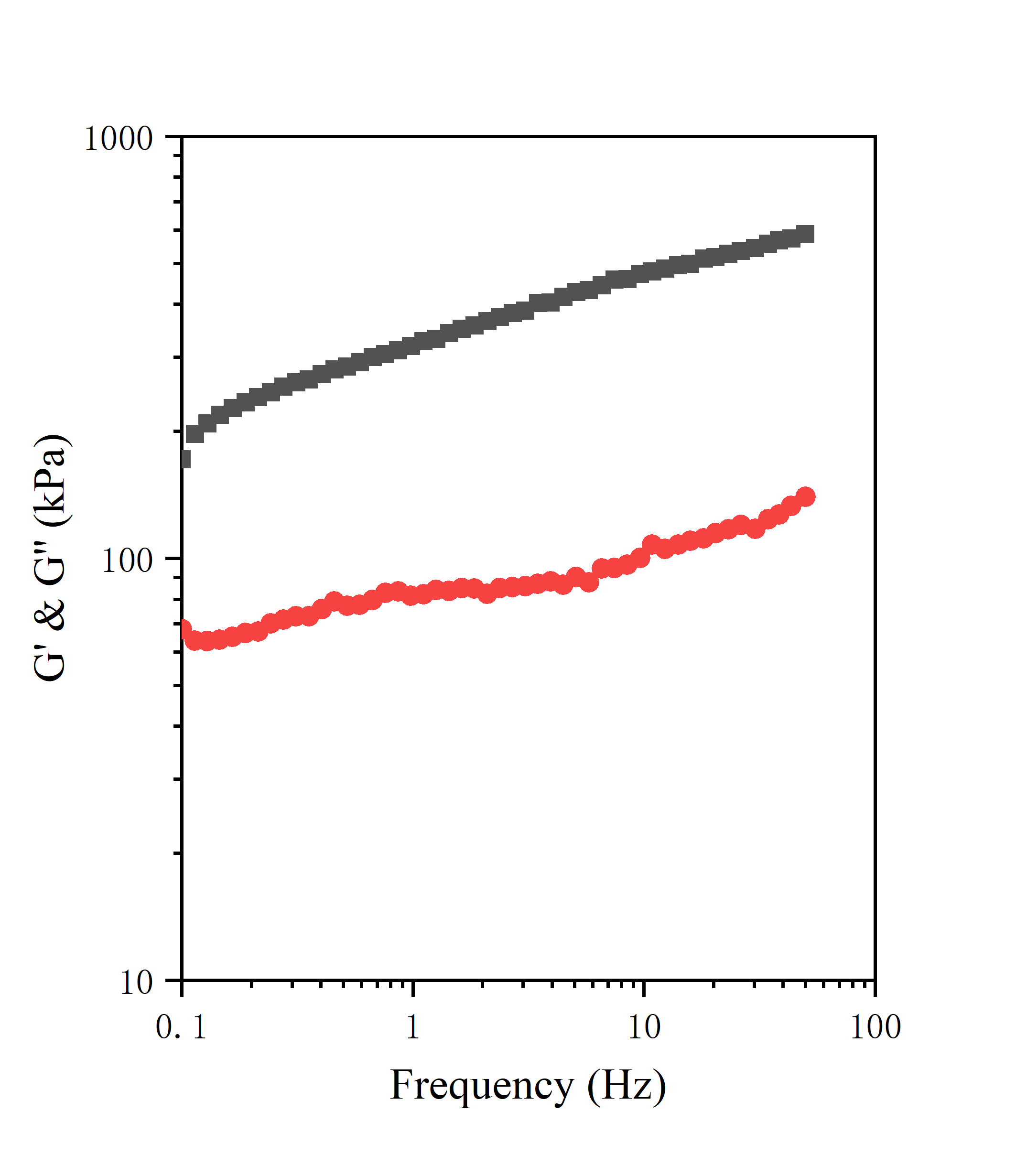

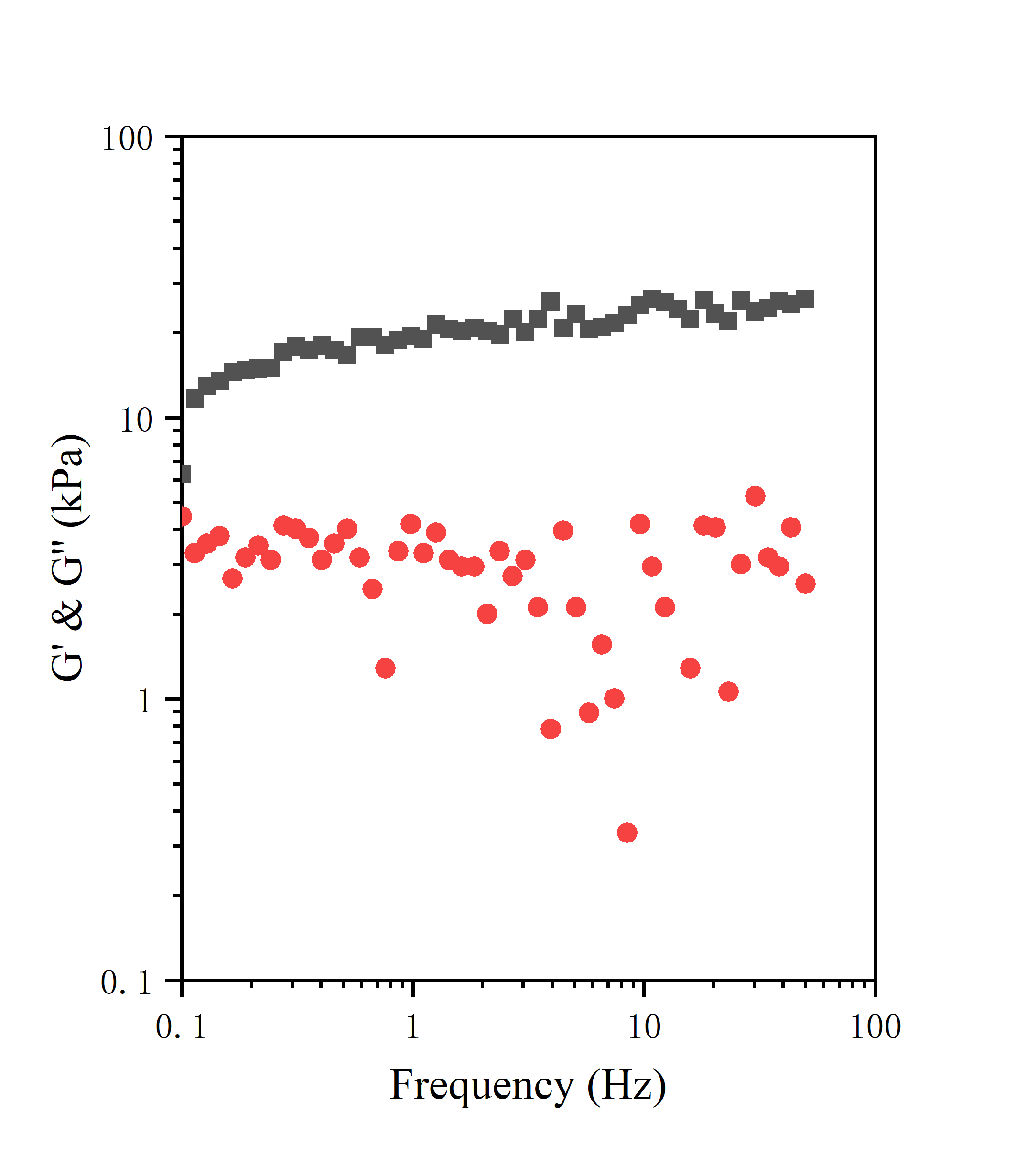

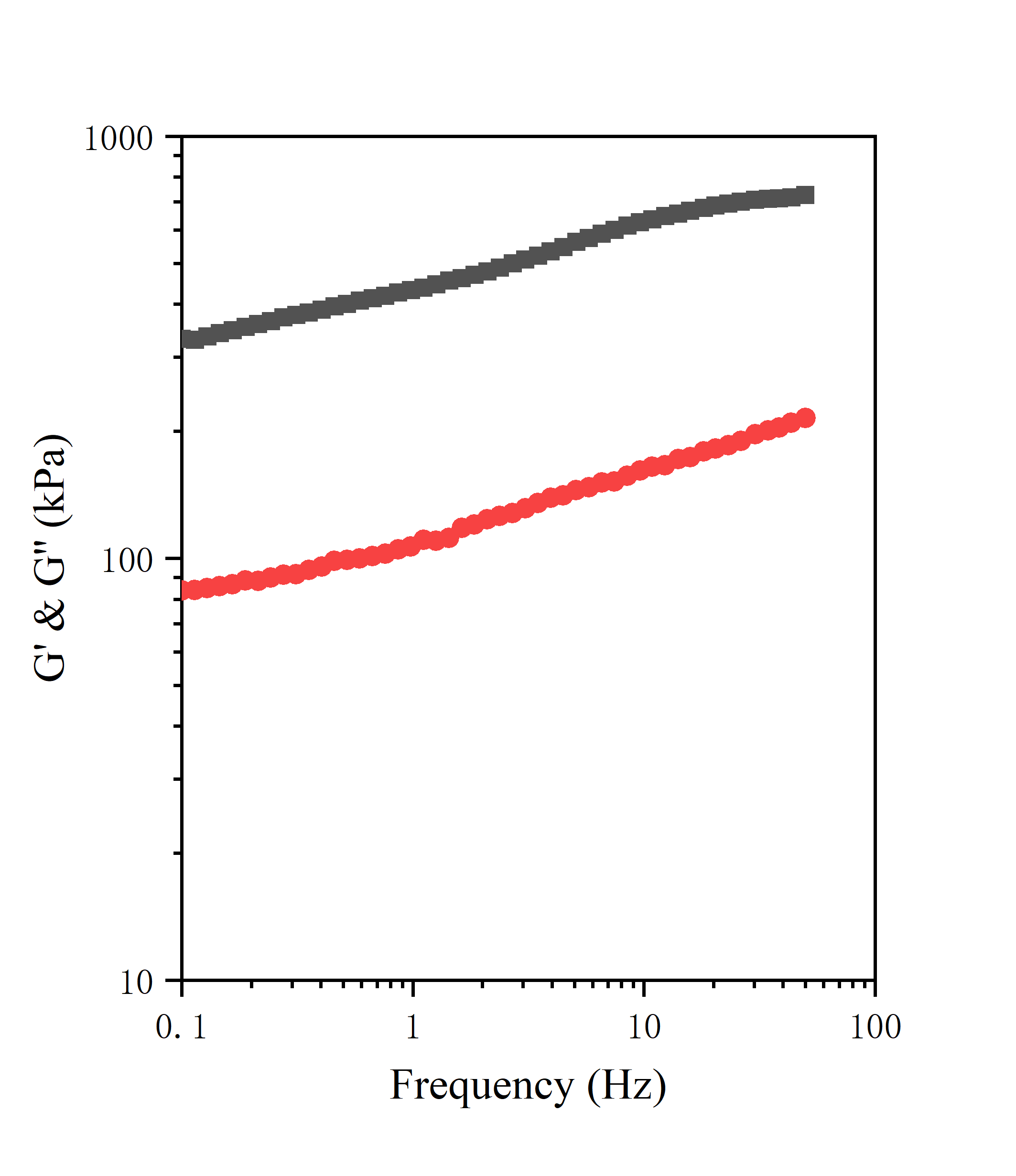


1%TG


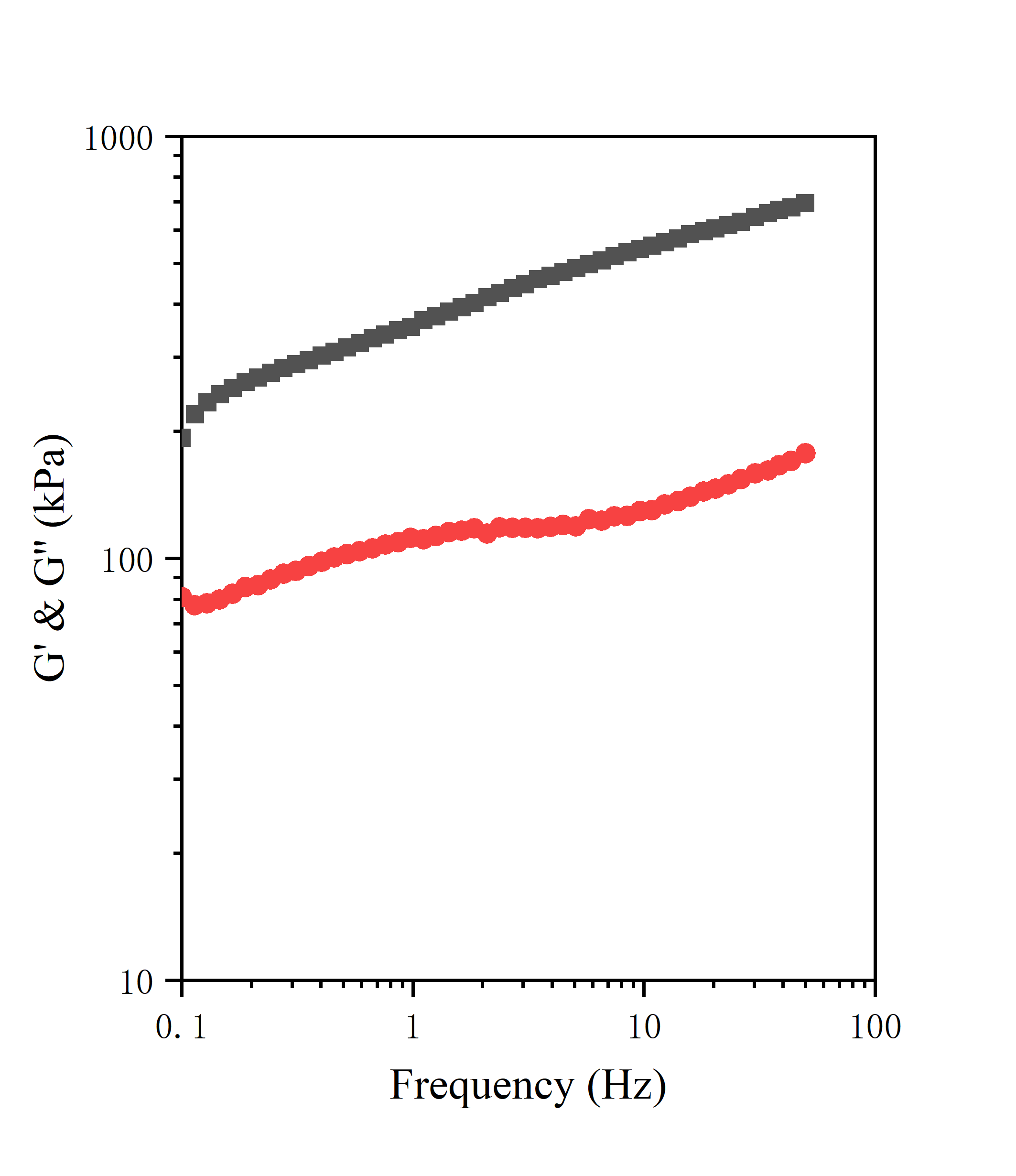

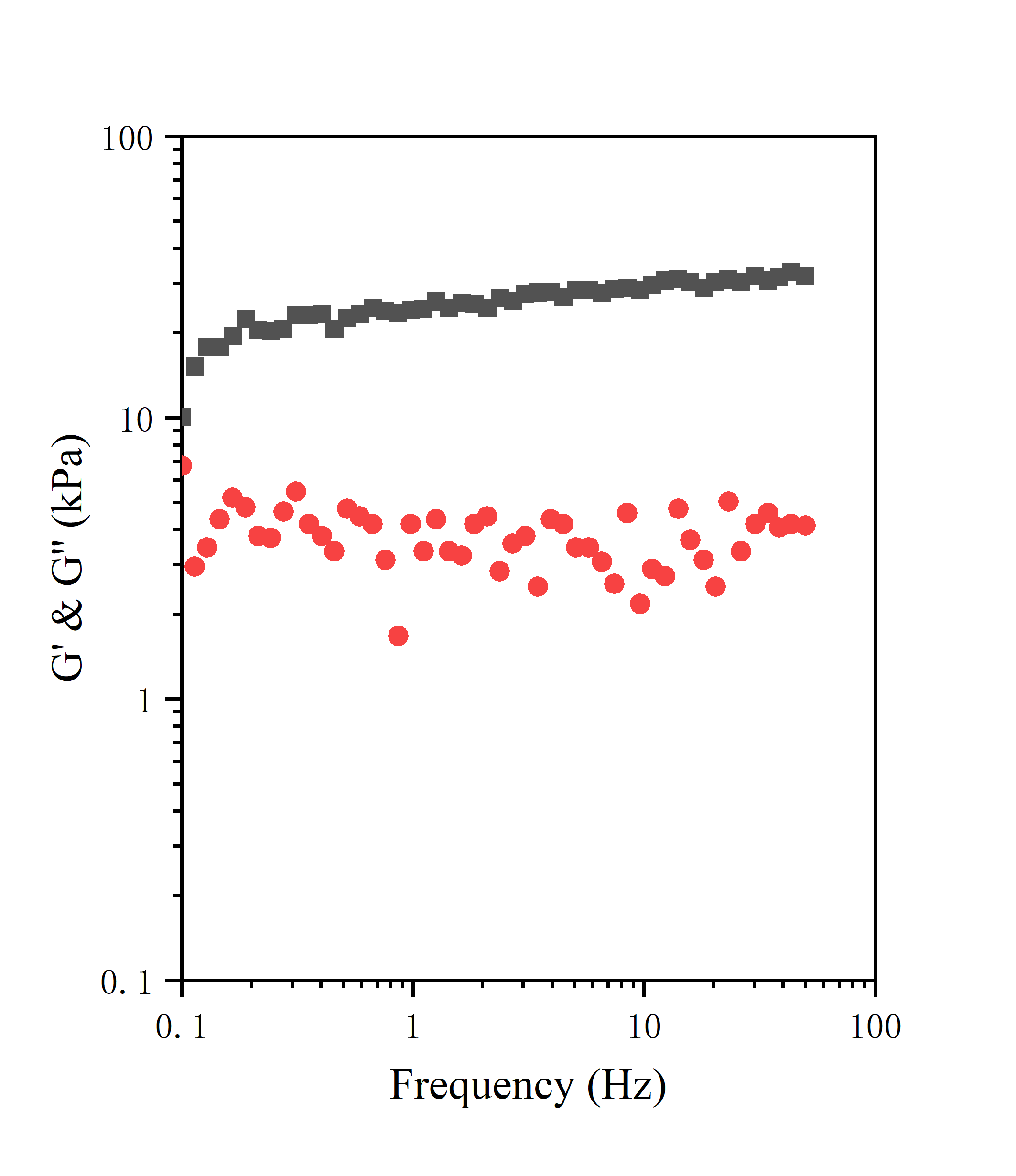

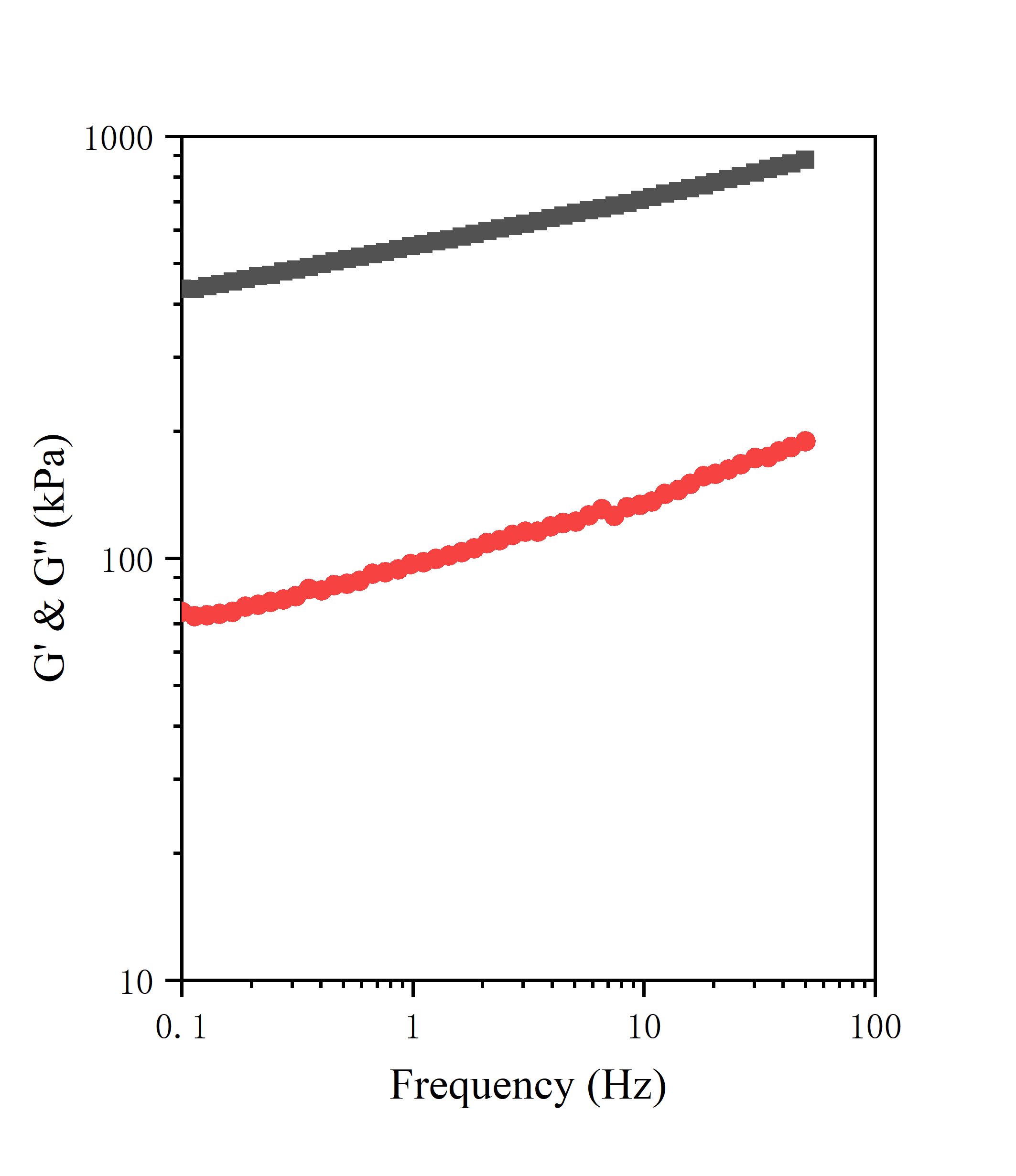


1.5%TG


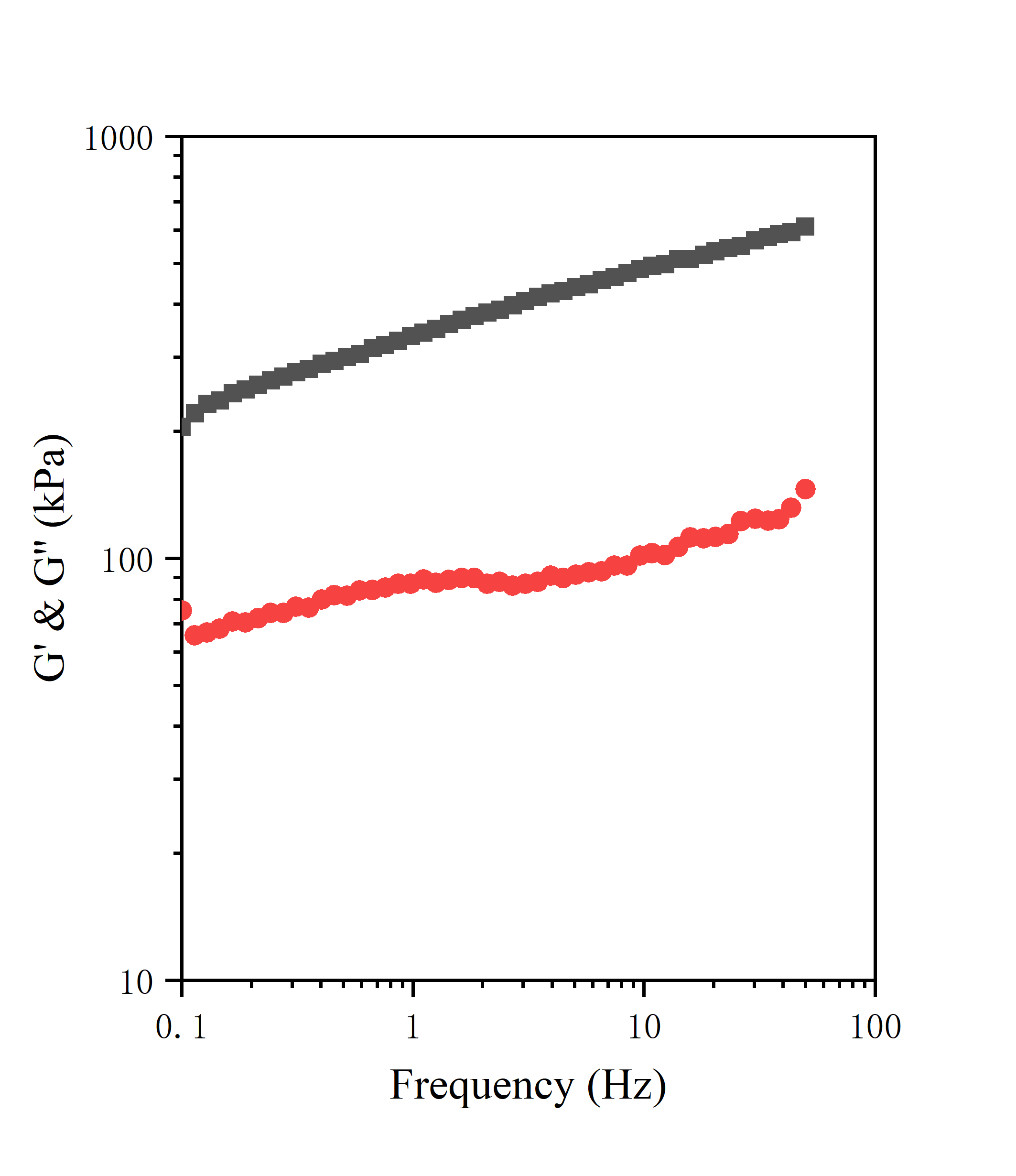

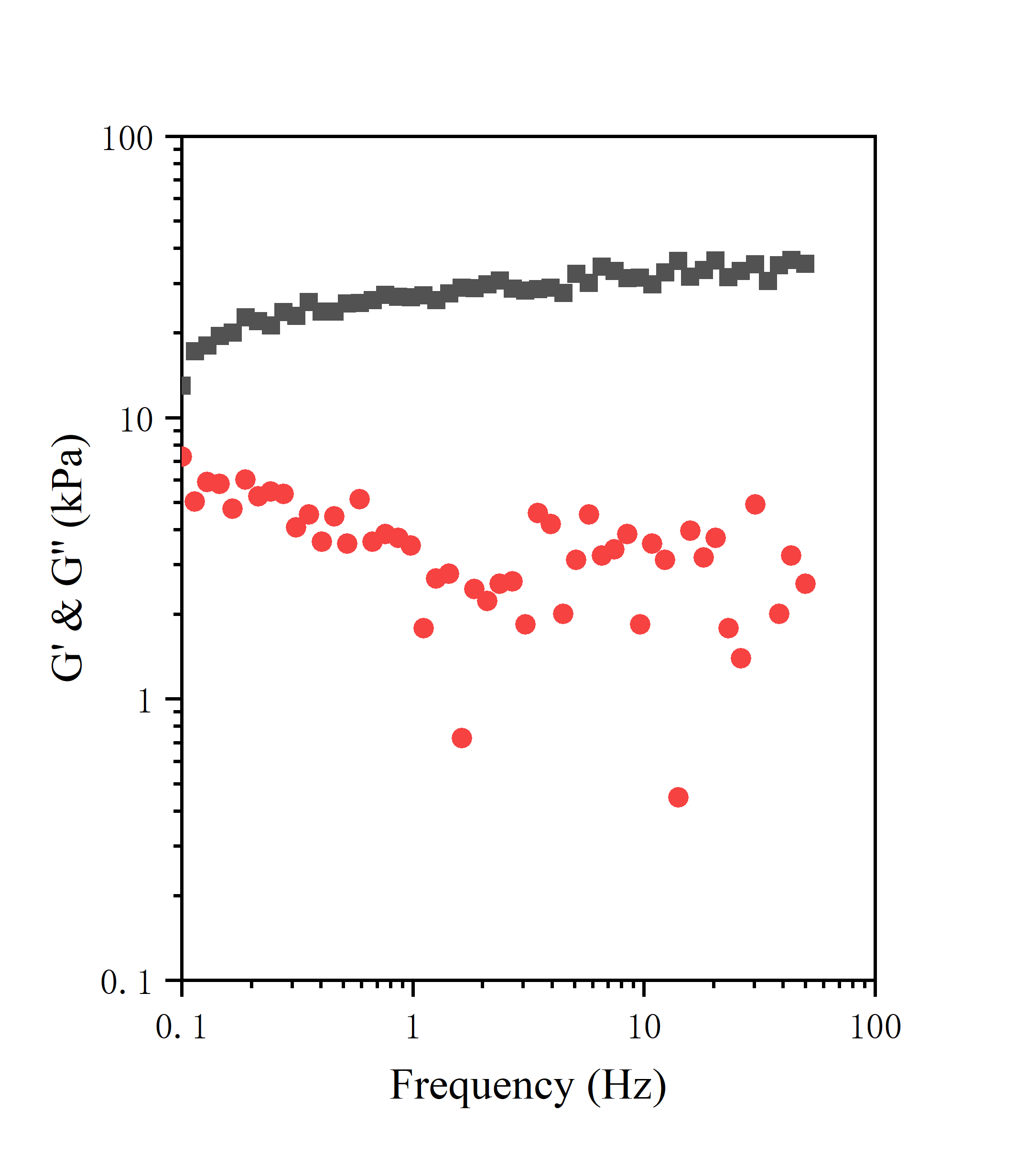

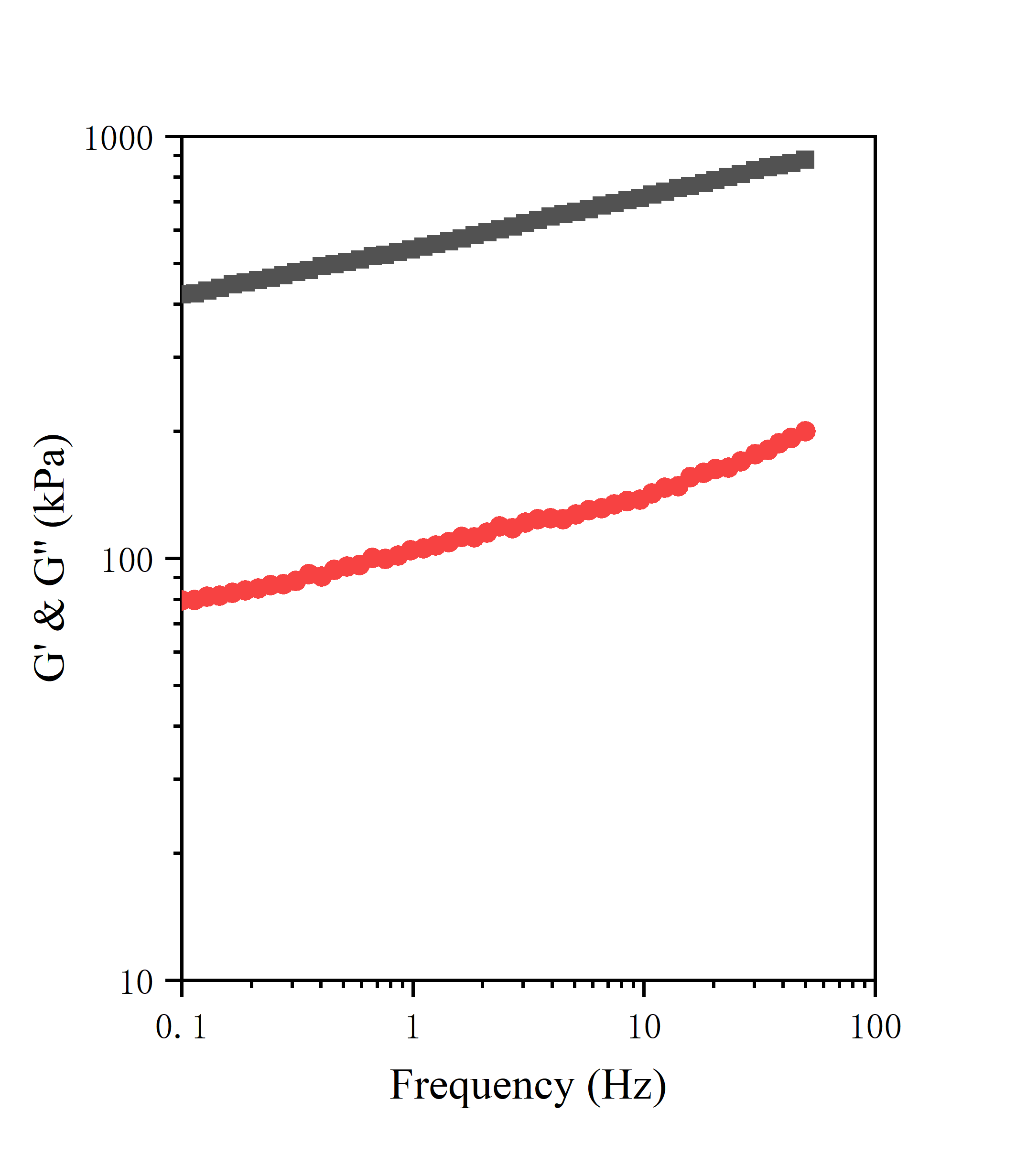


2%TG

**Table S1**

Frequency dependence of storage modulus of PPI with different concentrations of TG.

|  | **0%** | **0.25%** | **0.50%** | **1%** | **1.50%** | **2%** |
| --- | --- | --- | --- | --- | --- | --- |
| **30** °C | 0.383±0.007a | 0.376±0.007ab | 0.359±0.002cd | 0.347±0.010d | 0.365±0.008bc | 0.315±0.006e |
| **120** °C | 0.364±0.006a | 0.281±0.006b | 0.258±0.005c | 0.279±0.006b | 0.234±0.009d | 0.233±0.006d |
| **30-120-30** °C | 0.285±0.006a | 0.237±0.006c | 0.233±0.006cd | 0.259±0.007b | 0.213±0.005e | 0.224±0.006de |

Note: Values are expressed as the means and standard deviations of three measurements. Different letters in the same line indicate significant differences between groups (*P* < 0.05).
